# Comprehensive Analysis of the Nocardia cyriacigeorgica Complex Reveals Five Species-Level Clades with Different Evolutionary and Pathogenicity Characteristics

**DOI:** 10.1128/msystems.01406-21

**Published:** 2022-04-18

**Authors:** Shuai Xu, Ming Wei, Gang Li, Zhenpeng Li, Yanlin Che, Lichao Han, Wei Jia, Fang Li, Dan Li, Zhenjun Li

**Affiliations:** a State Key Laboratory for Infectious Disease Prevention and Control, National Institute for Communicable Disease Control and Prevention, Chinese Center for Disease Control and Prevention, Beijing, China; b Department of Infectious Diseases and Clinical Microbiology, Beijing Chao-yang Hospital, Capital Medical University, Beijing, China; c Medical Laboratory Center, General Hospital of Ningxia Medical University, Ningxia, China; d Key Laboratory of Medicine, Ministry of Education, School of Laboratory Medicine and Life Sciences, Wenzhou Medical University, Wenzhou, China; e School of Medical, Tibet University, Tibet, China; University of Delhi

**Keywords:** *Nocardia cyriacigeorgica* complex, comparative genomics, pan-genome, taxonomy, genetic diversity, evolution, pathogenicity

## Abstract

Nocardia cyriacigeorgica is a common etiological agent of nocardiosis that has increasingly been implicated in serious pulmonary infections, especially in immunocompromised individuals. However, the evolution, diversity, and pathogenesis of *N. cyriacigeorgica* have remained unclear. Here, we performed a comparative genomic analysis using 91 *N. cyriacigeorgica* strains, 45 of which were newly sequenced in this study. Phylogenetic and average nucleotide identity (ANI) analyses revealed that *N. cyriacigeorgica* contained five species-level clades (8.6 to 14.6% interclade genetic divergence), namely, the *N. cyriacigeorgica* complex (NCC). Further pan-genome analysis revealed extensive differences among the five clades in nine functional categories, such as energy production, lipid metabolism, secondary metabolites, and signal transduction mechanisms. All 2,935 single-copy core genes undergoing purifying selection were highly conserved across NCC. However, clades D and E exhibited reduced selective constraints, compared to clades A to C. Horizontal gene transfer (HGT) and mobile genetic elements contributed to genomic plasticity, and clades A and B had experienced a higher level of HGT events than other clades. A total of 129 virulence factors were ubiquitous across NCC, such as the *mce* operon, hemolysin, and type VII secretion system (T7SS). However, different distributions of three toxin-coding genes and two new types of *mce* operons were detected, which might contribute to pathogenicity differences among the members of the NCC. Overall, our study provides comprehensive insights into the evolution, genetic diversity, and pathogenicity of NCC, facilitating the prevention of infections.

**IMPORTANCE** *Nocardia* species are opportunistic bacterial pathogens that can affect all organ systems, primarily the skin, lungs, and brain. *N. cyriacigeorgica* is the most prevalent species within the genus, exhibits clinical significance, and can cause severe infections when disseminated throughout the body. However, the evolution, diversity, and pathogenicity of *N. cyriacigeorgica* remain unclear. Here, we have conducted a comparative genomic analysis of 91 *N. cyriacigeorgica* strains and revealed that *N. cyriacigeorgica* is not a single species but is composed of five closely related species. In addition, we discovered that these five species differ in many ways, involving selection pressure, horizontal gene transfer, functional capacity, pathogenicity, and antibiotic resistance. Overall, our work provides important clues in dissecting the evolution, genetic diversity, and pathogenicity of NCC, thereby advancing prevention measures against these infections.

## INTRODUCTION

The genus *Nocardia* is a Gram-positive, partially acid-fast, aerobic, catalase-positive actinobacterium commonly found in soil and aquatic habitats globally and is recognized as an opportunistic human and animal pathogen (1, 2). Currently, 120 *Nocardia* species have been described according to the *List of Prokaryotic Names with Standing in Nomenclature* (https://lpsn.dsmz.de/genus/nocardia), of which at least 54 can cause nocardiosis (3). The infection mainly causes mycetoma, chronic bronchitis, and brain abscesses (4–6). Notably, it can progress into a systemic condition by hematogenous dissemination, and once it occurs, mortality and morbidity dramatically increase (7, 8).

Nocardia cyriacigeorgica is one of the most prevalent etiological agents of nocardiosis and most likely to cause severe pulmonary and disseminated infections (9–14). Despite the clinical relevance and importance of this pathogen, our current understanding of the pathogenicity of *N. cyriacigeorgica* is limited. In addition, several studies have reported a high degree of heterogeneity within *N. cyriacigeorgica* strains, which comprised three subgroups based on PCR and multilocus sequence analysis (MLSA) approaches (15–20). A recent report in our group using 20 genome assemblies proposed that the subgroups of *N. cyriacigeorgica* should be regarded as closely related species (21). However, an in-depth comparative genomics analysis has yet to be conducted to resolve questions regarding the genetic variation and evolution of *N. cyriacigeorgica*. Indeed, much of our understanding of *N. cyriacigeorgica* relies on the GUH-2 genome (22).

Here, we present a comprehensive analysis of 91 *N. cyriacigeorgica* strains, representing the first comparative genomics study of this group. These data sets and analyses provide new insights into the phylogenetic relationships, genetic diversity, evolutionary trends, and pathogenicity potential of *N. cyriacigeorgica*.

## RESULTS

### Genomic characteristics of *N. cyriacigeorgica*.

**FIG 1:**
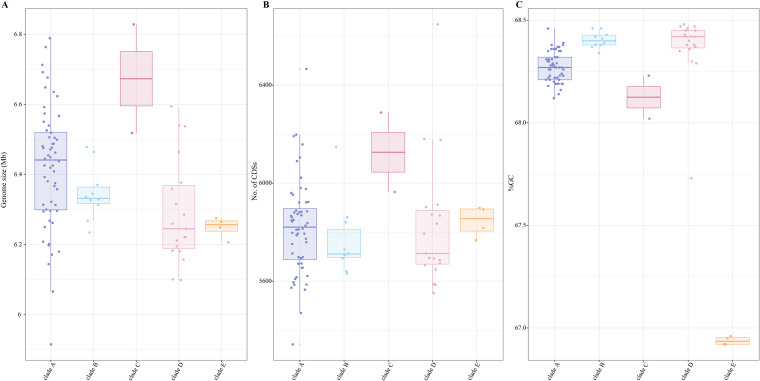
Genome size, number of CDSs, and GC content of genomes analyzed in this study. (A) Genome size, (B) number of CDSs, and (C) GC content. The genomes are colored based on phylogenetic clades as defined in Fig. 2B.

We investigated 91 *N. cyriacigeorgica* genomes that passed strict quality control, spanning 46 years from 1975 to 2020, thereby providing the first systematic comparative genomics study of *N. cyriacigeorgica* (see Table S1 in the supplemental material). The source from which most isolates were obtained was human sputum, followed by bronchoalveolar lavage fluid, suggesting that most strains cause pulmonary infection. All genomes, including 45 newly sequenced and 46 publicly available genomes, were identified as *N. cyriacigeorgica* based on an analysis of 16S rRNA gene sequences (identity, >99%). The genome sizes of the 91 genomes were approximately 6.38 Mb (ranging from 5.92 to 6.83 Mb), with the predicted number of coding sequences (CDSs) ranging from 5,342 to 6,648. Notably, the GC content of these genomes showed some variation, with an average value of 68.24% (range, 66.92% to 68.48%): of these, four genomes seem unusual, having GC content lower by 1% than those of other assemblies (Fig. 1).

10.1128/msystems.01406-21.6 TABLE S1 Genome sequences of strains used in this study. Download Table S1, PDF file, 0.1 MB. Copyright © 2022 Xu et al. 2022 Xu et al. https://creativecommons.org/licenses/by/4.0/ This content is distributed under the terms of the Creative Commons Attribution 4.0 International license.

### Phylogenetic and average nucleotide identity analysis reveals five species-level clades of *N. cyriacigeorgica* complex.

**FIG 2:**
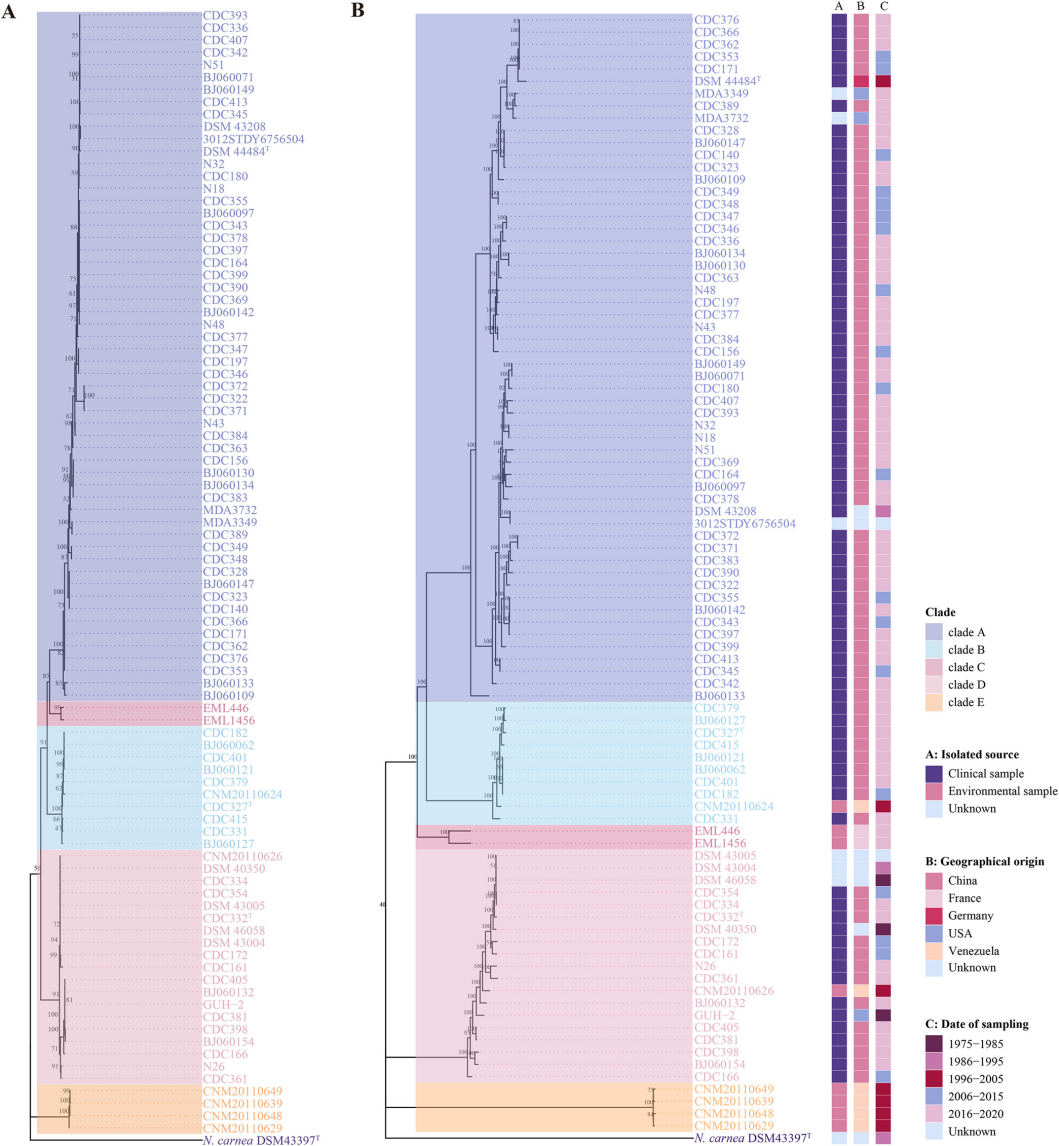
Phylogenetic trees of 91 genome assemblies of *N. cyriacigeorgica* complex. (A) The *dapb1*-based phylogenetic tree. (B) The phylogenomic tree constructed based on the concatenation of the nucleotide sequences of 2,935 single-copy core genes. The isolation source, geographical origin, and date of sampling are shown. Each tree was built using the maximum likelihood method, with 1,000 bootstrap replicates. The genome sequence of *N. carnea* DSM 43397^T^ was used as an outgroup, and its branch length was shortened for better visualization. Bootstrap values are indicated on the nodes, and five main phylogenetic clades are highlighted in different colors.

An initial phylogenetic tree was constructed using the *dapb1* gene sequence of 91 *N. cyriacigeorgica* strains. This gene has been previously reported to reliably infer phylogenetic relationships for the genus *Nocardia* (21). The genome of Nocardia carnea DSM 43397^T^ was selected as an outgroup for the phylogenetic analysis due to its close evolutionary relationship to *N. cyriacigeorgica* (21). According to the topology, the *dapb1* gene tree revealed the presence of five main groups (Fig. 2A). To further assess the genetic relatedness of strains, we constructed a high-quality maximum likelihood (ML) phylogenetic tree of *N. cyriacigeorgica* using 2,935 single-copy core genes (Fig. 2B). In general, except for the difference in the relative positions of the clades and strain order within these clades, the two trees showed similar topological structures, resulting in the identification of five distinct clades. Clade A harbored three subclades and contained the majority of *N. cyriacigeorgica* genomes (including strain DSM 44484^T^) (Fig. 2B). Clades B and D were composed of 10 and 19 strains, respectively, most of which were isolated from humans. The smallest clade (C) contained two genomes, EML446 and EML1456, isolated from infiltration basin urban sediments. Finally, clade E consists of environmental strains isolated from soil, with longer branch lengths than the rest of the branches between the clades. Interestingly, the members of clade E were also those with decreased GC content (Fig. 1), suggesting its more distant genetic relationship to other strains.

**FIG 3:**
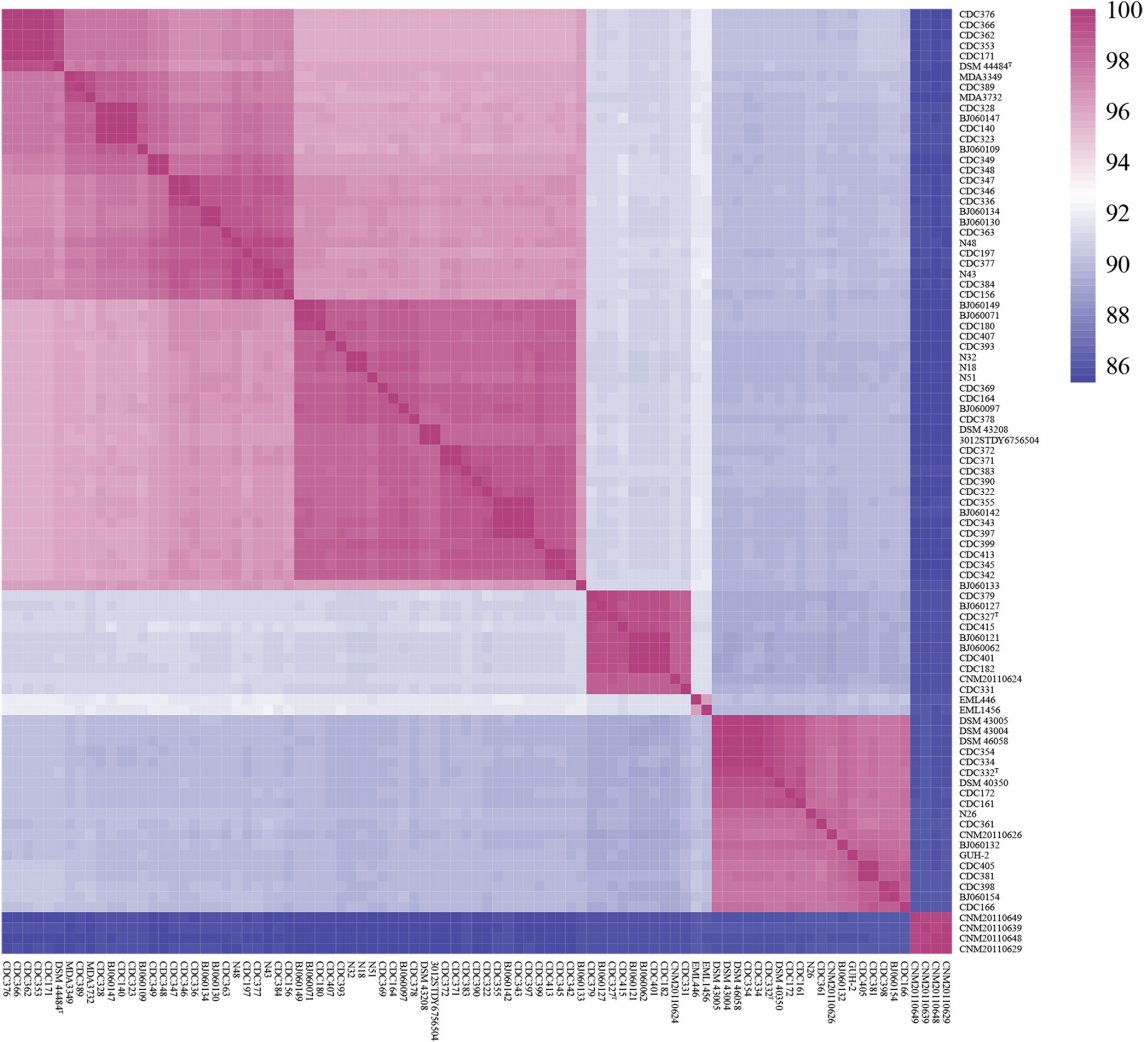
Heat map of pairwise ANI values for 91 genome assemblies of *N. cyriacigeorgica* complex.

Further average nucleotide identity (ANI) analysis also supported the relationships defined by phylogeny (Fig. 2B). Currently, a 95% to 96% ANI value between pairwise genomes is considered to indicate they belong to the same species (23). The ANI values of different clades ranged from 85.32% to 91.38% (Fig. 3), below the species threshold, suggesting that each clade consists of distinct species from the other clades. Conversely, the intraclade ANI values all exceeded the 95% species-level threshold (i.e., ≥95.62% in clade A, ≥98.53% in clade B, ≥96.27% in clade C, ≥97.61% in clade D, and ≥99.66% in clade E), suggesting that each clade consists of a single species. Altogether, these results robustly support the division of the species *N. cyriacigeorgica* into an emended species (clade A) and four novel species (clades B to E). Thus, we propose the name Nocardia cyriacigeorgica complex (NCC) comprised of these five species.

### Phenotypic characterization of new species.

Because no strains of clades C and E were available in our laboratory, we selected isolates from each of the two clades B and D (clade B, strain CDC327^T^; clade D, strain CDC332^T^) to provide detailed phenotypic characterization. Two isolates were able to grow at 28°C to 40°C, at pH 6.0 to 10.0, and in the presence of 0 to 6.5% NaCl (wt/vol). The optimal growth conditions of the isolates were 37°C, pH 7.0, and 0% NaCl.

The biochemical characteristics of strains CDC327^T^ and CDC332^T^ were determined using API 50 CH and API ZYM and compared with those of their phylogenetically close neighbor (clade A, *N. cyriacigeorgica* DSM 44484^T^). The two isolates could utilize glycerol (only strain CDC332^T^), ribose (only CDC332^T^), l-xylose (weakly), adonitol (weakly), β-methyl-d-xylopyranoside (only CDC332^T^), fructose (weakly), mannose (weakly), rhamnose (only CDC327^T^), mannitol (only CDC327^T^), esculin, and d-turanose (only CDC327^T^) as sole carbon sources, but not erythritol, d-arabinose, l-arabinose, d-xylose, calactose, glucose, sorbose, melampyrin, *myo*-inositol, sorbitol, α-methyl-d-mannopyranoside, α-methyl-d-glucopyranoside, *N*-acetylglucosamine, amygdaloside, arbutin, salicin, cellobiose, maltose, lactose, melibiose, sucrose, trehalose, inulin, melezitose, raffinose, starch, glycogen, xylitol, gentiobiose, d-lyxose, d-tagatose, d-fucose, l-fucose, d-arabinose, l-arabinose, potassium gluconate, potassium 2-ketogluconate, and potassium 5-ketogluconate. Positive activity was detected for alkaline phosphatase, esterase (C_4_), esterase lipase (C_8_), leucine arylamidase, trypsin (only CDC332^T^, weakly positive), valine arylamidase (weakly positive for CDC332^T^), acid phosphatase, naphthol-AS-BI-phosphohydrolase, β-galactosidase, β-glucosidase, and β-d-glucosidase; negative activity was found for lipase (C_14_), cystine arylamidase, chymotrypsin, α-galactosidase, β-glucuronidase, *N*-acetylglucosamine, α-mannosidase, and β-fucosidase. The differential biochemical properties of strains CDC327^T^ and CDC332^T^ and related strain *N. cyriacigeorgica* DSM 44484^T^ are summarized in Table S2 in the supplemental material.

10.1128/msystems.01406-21.7 TABLE S2 Differential phenotypic properties between isolates CDC327^T^ and CDC332^T^ from their closely related species *N. cyriacigeorgica* DSM 44484^T^. Download Table S2, PDF file, 0.07 MB. Copyright © 2022 Xu et al. 2022 Xu et al. https://creativecommons.org/licenses/by/4.0/ This content is distributed under the terms of the Creative Commons Attribution 4.0 International license.

We then evaluated the cellular fatty acid profiles of these two novel species to identify their chemotaxonomic characteristics. The major fatty acids of isolate CDC327^T^ were C_16:0_ (28.67%) and summed features 3 (C_16:1_ω6c/C_16:1_ω7c, 23.66%), while isolate CDC332^T^ had summed features 3 (C_16:1_ω6c/C_16:1_ω7c, 33.04%) and C_16:0_ (25.40%). The detailed cellular fatty acid profiles of strains CDC327^T^, CDC332^T^, and the reference strain *N. cyriacigeorgica* DSM 44484^T^ are presented in Table S3 in the supplemental material.

10.1128/msystems.01406-21.8 TABLE S3 Major cellular fatty acid contents of CDC327^T^ and CDC332^T^ and their closely related species *N. cyriacigeorgica* DSM 44484^T^. Download Table S3, PDF file, 0.06 MB. Copyright © 2022 Xu et al. 2022 Xu et al. https://creativecommons.org/licenses/by/4.0/ This content is distributed under the terms of the Creative Commons Attribution 4.0 International license.

### Description of *Nocardia ningxiensis* sp. nov.

*ningxiensis* (ning.xi.en′sis. N.L. fem. adj. *ningxiensis*, pertaining to Ningxia, where the type strain was isolated). The type strain is CDC327 (JCM 34961^T^ = GDMCC 4.210^T^), isolated from the sputum of an 80-year-old male patient in the Ningxia Hui Autonomous Region of China. The genome accession number is CRX204748 in the Genome Sequence Archive (GSA). The genome size is 6.23 Mb, with a GC content of 68.46%. It belongs to clade B.

### Description of *Nocardia yinchuanensis* sp. nov.

*yinchuanensis* (yin.chuan.en′sis. N.L. fem. adj. *yinchuanensis*, pertaining to Yinchuan, where the type strain was isolated from). The type strain is CDC332 (JCM 34962^T^ = GDMCC 4.212^T^), isolated from an alveolar lavage fluid of a 40-year-old male patient in Yinchuan, Ningxia Hui Autonomous Region of China. The genome accession number is CRX204747 in the GSA. The genome size is 6.21 Mb, with a GC content of 68.46%. It belongs to clade D.

### Pan-genome analysis highlights functional differences among five clades of NCC.

**FIG 4:**
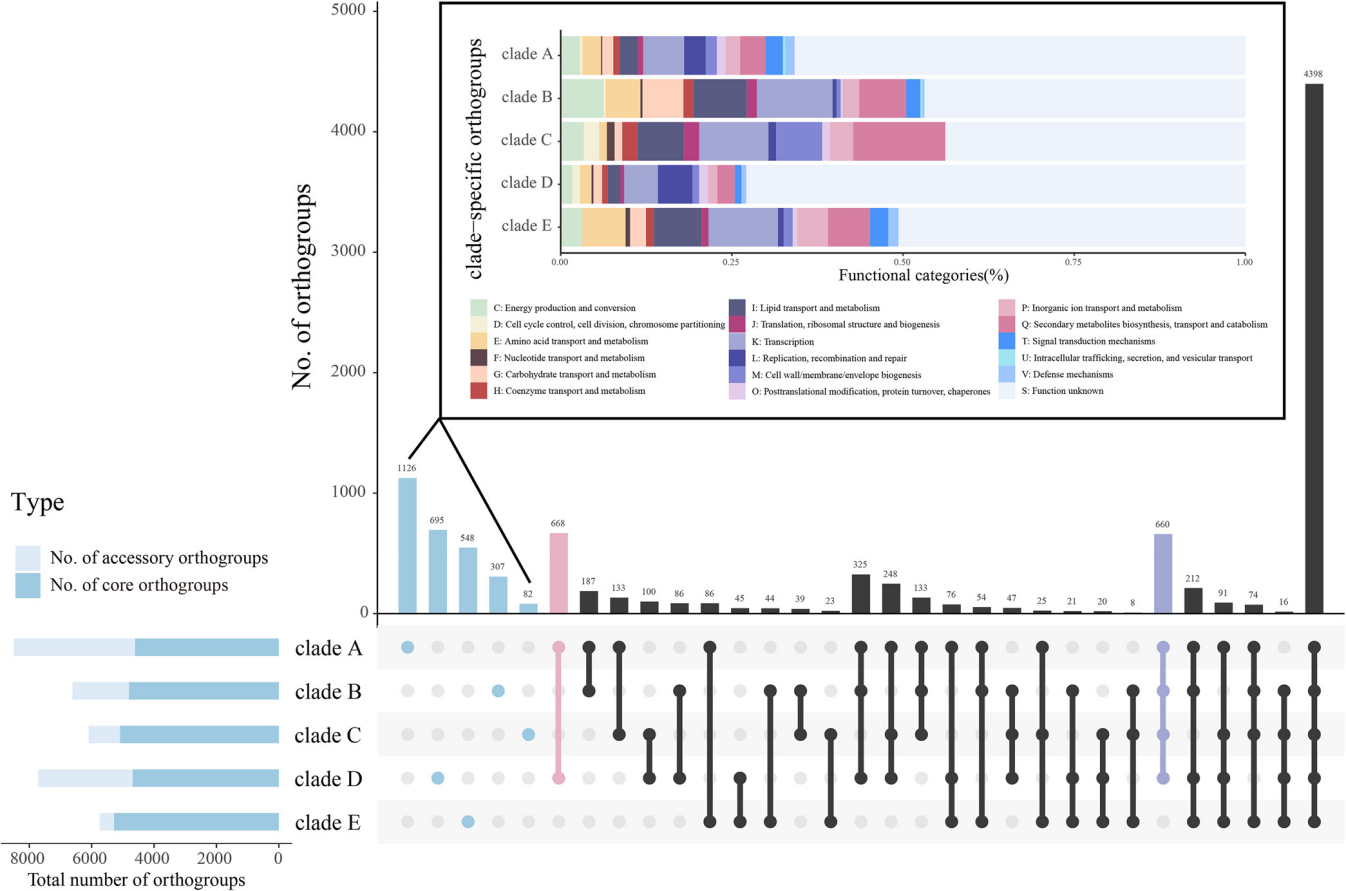
Distribution of the number of orthogroups in the *N. cyriacigeorgica* complex. Horizontal bars indicate the number of core and accessory orthogroups in the five clades. Vertical bars represent the number of clade-specific orthogroups or shared orthogroups between the five clades. Clade-specific orthogroups are indicated in blue, and their distributions of functional categories based on eggNOG annotation are shown in the inset. The orthogroups unique to clades A and D are indicated in pink, whereas the orthogroups unique to clades A to D are in purple.

To further characterize the genomic composition of NCC, we performed pan-genome construction and analysis of 91 genomes. These strains contained 10,577 different orthogroups (defined as the group of genes descended from a single gene in the last common ancestor of all the species) using OrthoFinder (24). Of these 4,104 shared by 96% of the strains, were identified as core genes, while the remaining 6,473 were identified as accessory orthogroups. We next compared the distributions of orthogroups between the different clades (Fig. 4). The size of core genomes of each clade varied between 4,615 (clade A) and 5,279 (clade E). Clade E showed the lowest number of accessory orthogroups (462), probably due to a small number of available genomic assemblies. The clade with the highest number of clade-specific gene families was clade A (1,126), followed by clade D (695) and clade E (548). Moreover, clades A and D shared 668 uniquely shared orthogroups, which greatly outnumbered other clades. Of note, clades A to D shared more unique orthogroups (660), further reflecting the genetic distance of clade E from the other clades.

We further characterized the functions of all clade-specific orthogroups based on eggNOG database annotations (Fig. 4). High proportions of orthogroups were classified as “function unknown,” indicating that further functional research is necessary. The relative distribution of functional categories for clade-specific orthogroups was uneven; for instance, clade B isolates possessed more unique genes involved in crucial metabolic functions, especially those related to amino acid, carbohydrate, and lipid metabolism. Genes annotated as “secondary metabolites biosynthesis, transport, and catabolism” were prominently in clade C. Moreover, clades A and E harbored a higher percentage of unique genes enriched in defense mechanisms than the other clades. These results indicate that some unique genes within NCC members varied in functions to adapt to environmental changes.

A principal-coordinate analysis (PCoA) based on the presence and absence profiles of orthogroup showed the formation of five groups, corresponding to the phylogenetic clades (see Fig. S1 in the supplemental material). To obtain more insights into the overall functional capacity of the strains, we mapped all orthogroups to the eggNOG database. Of the 10,577 orthogroups, 48.9% were under “function unknown,” meaning that almost half of the orthogroups in the NCC have yet to be experimentally characterized. Next, we applied PCoA to recover the differences in gene content of the genomes for 18 functional categories.

10.1128/msystems.01406-21.1 FIG S1 PCoA based on the presence of orthogroups. Each point represents a genome colored by the clade it belongs to. Download FIG S1, PDF file, 0.1 MB. Copyright © 2022 Xu et al. 2022 Xu et al. https://creativecommons.org/licenses/by/4.0/ This content is distributed under the terms of the Creative Commons Attribution 4.0 International license.

**FIG 5:**
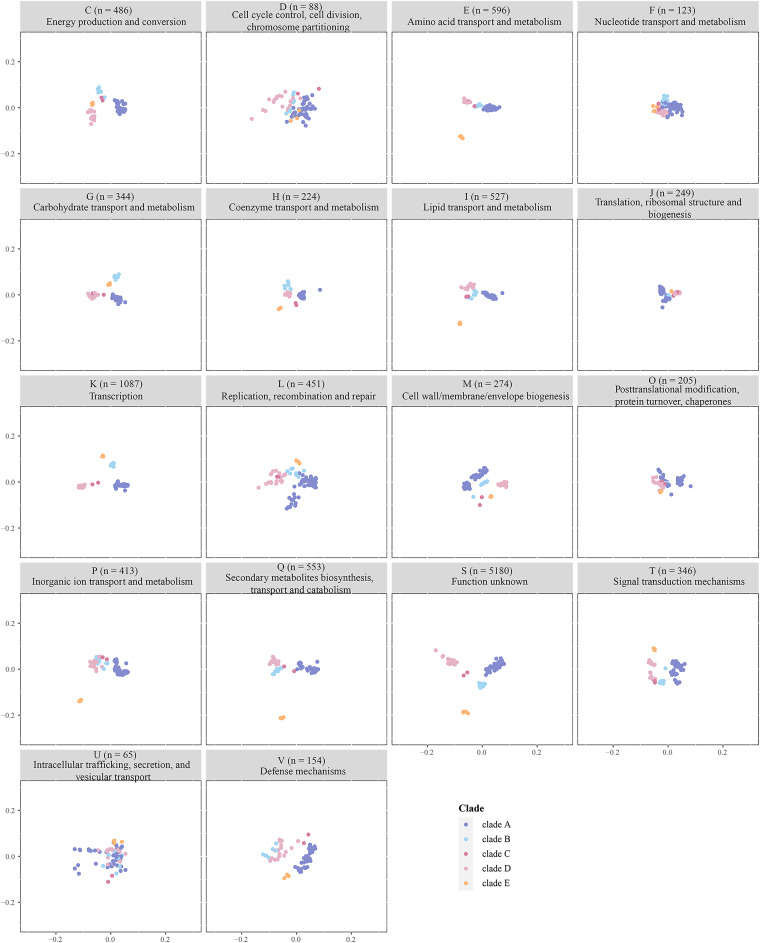
PCoA plots of predicted functional classification in each clade based on eggNOG annotation. Each point represents a genome that is colored based on its clade.

Figure 5 depicts that interclades have obvious or partial overlapping orthogroups that are related to “D: cell cycle control, cell division, chromosome partitioning,” “F: nucleotide transport, and metabolism,” “J: translation, ribosomal structure, and biogenesis,” “L: replication, recombination, and repair,” “O: posttranslational modification, protein turnover, and chaperones,” and “U: intracellular trafficking, secretion, and vesicular transport,” all of which represent essential bacterial functions. In contrast, clades B to D cluster separately from clades A and E on the function “P: inorganic ion transport and metabolism.” Clades B and C cluster separately from other clades based on the function “V: defense mechanisms.” More significantly, we observed that half of the functional categories (C, E, G, H, I, K, M, Q, and T) showed a clear separation organized by evolutionary relationships. These results indicate the presence of significant functional differences among clades at the genomic level.

### Distinct evolutionary rates in the NCC core genomes based on selective pressure analysis.

To gain insights into the evolutionary dynamics of the NCC core genome, we estimated the nonsynonymous (*K_a_*) and synonymous (*K_s_*) substitution rates of 2,935 single-copy core genes. A *K_a_*/*K_s_* ratio of >1 indicates positive selection, while a *K_a_*/*K_s_* of <1 indicates purifying selection. A *K_a_*/*K_s_* ratio of 1 indicates neutral selection (25). In general, more conserved genes have lower *K_a_*/*K_s_* values. We observed that all single-copy core genes were under strong purifying selection, with a median *K_a_*/*K_s_* ratio of 0.083, indicating their low level of genetic divergence as essential genes.

**FIG 6:**
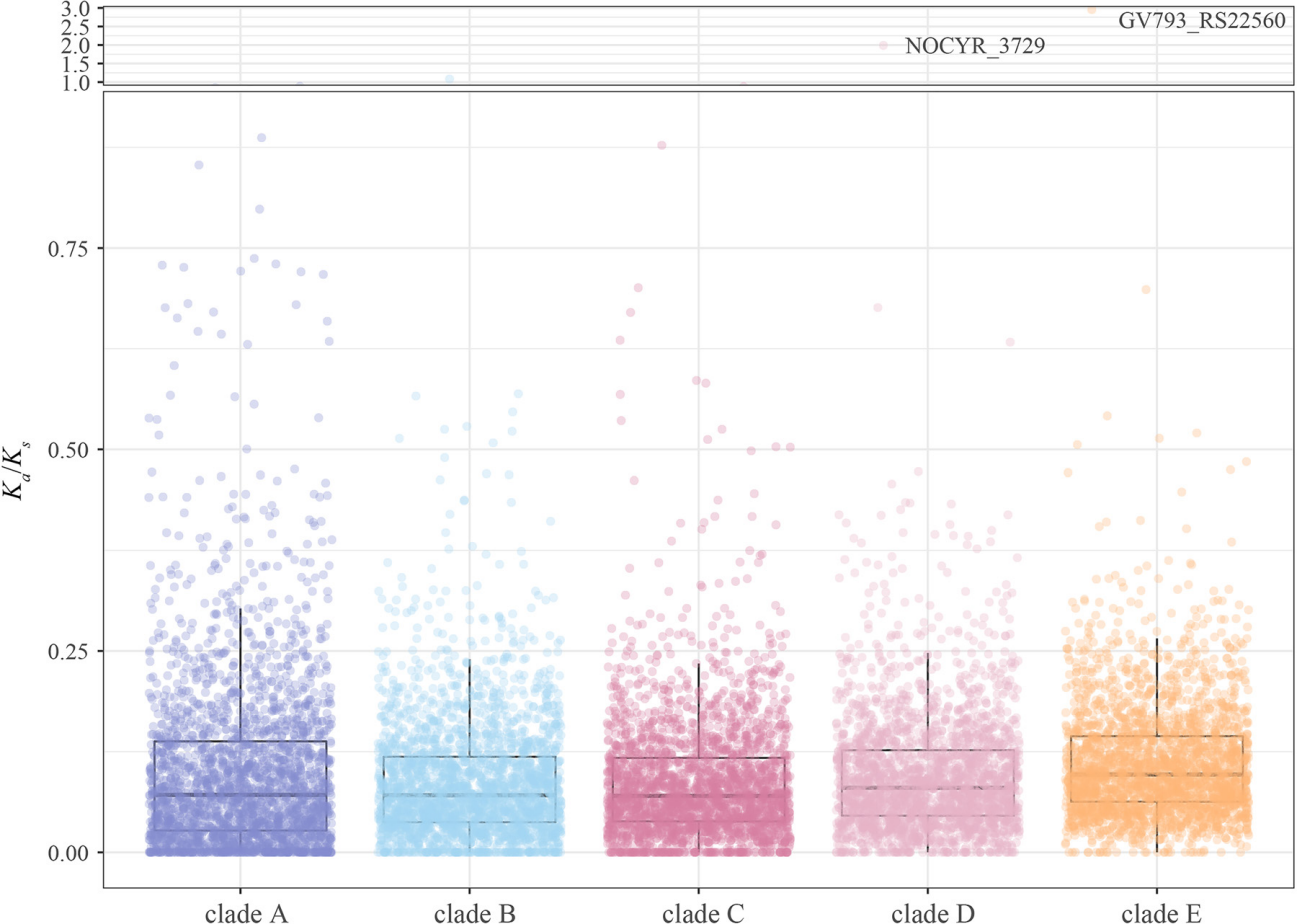
Selective pressure on 2,935 single-copy core genes of five clades in the *N. cyriacigeorgica* complex.

To further determine whether there were different evolutionary rates across NCC, we compared the selective pressure signals of the five clades. Although the median for five clades was below 1, the *K_a_*/*K_s_* values for clades E and D were significantly higher than those for the rest of the clades (*P* < 0.001, Wilcoxon’s two-sided test) (Fig. 6). This suggests that the single-copy core genes of clades E and D may be under relatively relaxing purifying selection or elevated diversifying selection, compared to those of other clades. Notably, the *yajC* gene in clade B had a *K_a_*/*K_s_* value of 1.08, indicating neutral selection. The genes NOCYR_3729 (*K_a_*/*K_s_* = 1.990) in clade D and GV793_RS22560 (*K_a_*/*K_s_* = 2.956) in clade E appeared to be under positive selection. These positively selected mutations may confer additional advantages for survival under specific environmental conditions, which warrant further investigation.

### Horizontal gene transfer and mobile genetic elements contribute to genomic plasticity.

**FIG 7:**
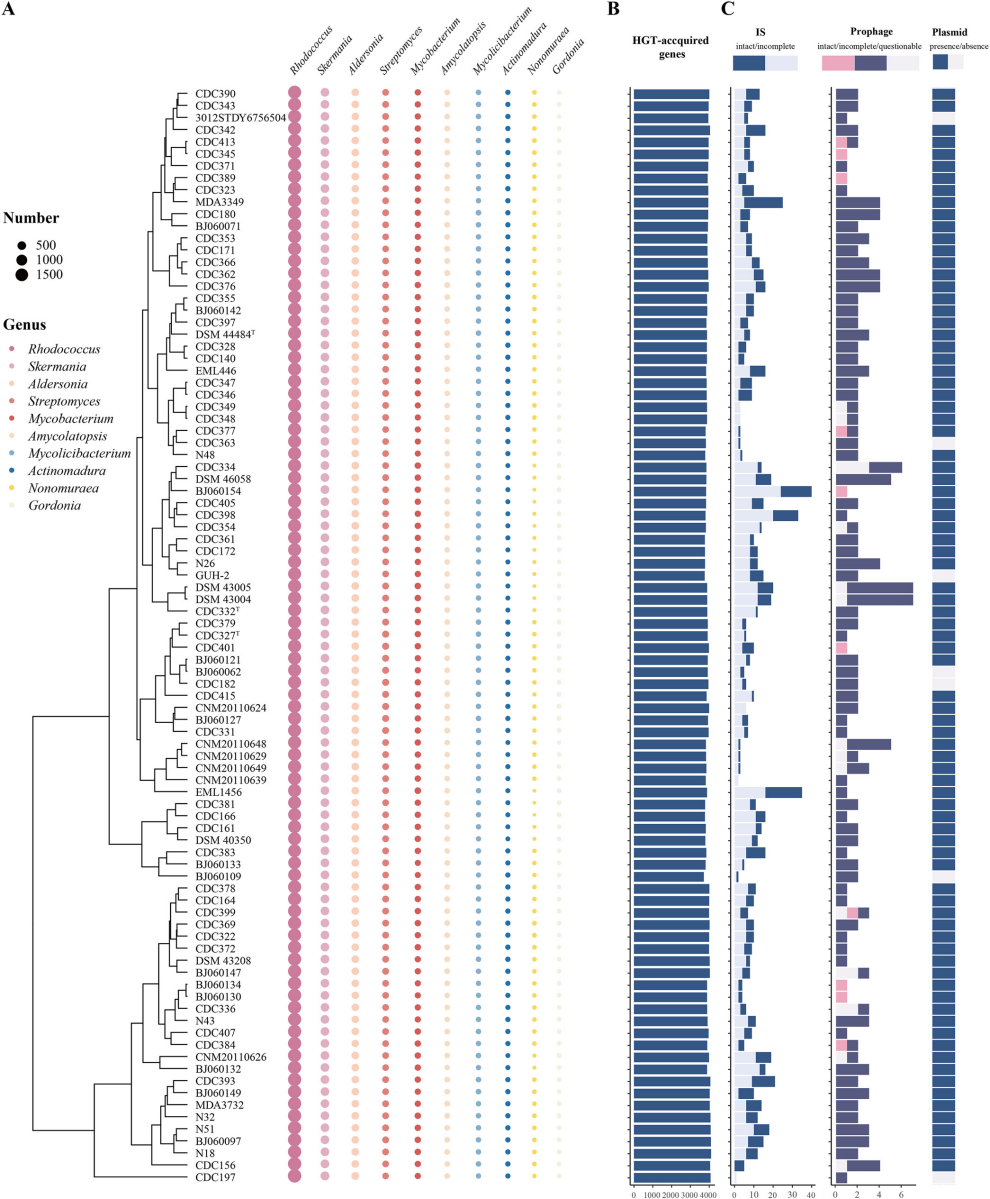
Distribution of horizontally transferred genes and MGEs in the *N. cyriacigeorgica* complex. (A) The top 10 potential donor bacterial genera provided donor genes for HGT. (B) The number of HGT-acquired genes in each genome. (C) The distribution of MGEs.

Horizontal gene transfer (HGT) is the physical process of transferring and integrating foreign genes into the genomes of recipients by transduction, conjugation, or transformation. This procedure enables the host to acquire new properties, affecting their metabolism, pathogenicity, antibiotic resistance, and adaptation to dynamic environments (26, 27). Here, we searched for potential HGT-acquired genes in the NCC genomes and tracked the potential donor taxa. A total of 6,082 gene families were identified that were potentially acquired by HGT, accounting for 57.5% of the gene families of the pan-genome and suggesting a high degree of genomic plasticity (see Fig. S2B in the supplemental material). These HGT genes were mainly involved in “S: function unknown” (1,837 genes [30.2%]), “K: transcription” (765 genes [12.5%]), “E: amino acid transport and metabolism” (526 genes [8.6%]), “I: lipid transport and metabolism” (455 genes [7.4%]), “C: energy production and conversion” (455 genes [6.8%]), and “Q: secondary metabolite biosynthesis, transport, and catabolism” (409 genes [6.7%]) (Fig. S2A). We then determined the potential HGT events between NCC and other bacteria. In general, a total of 297 potential donor taxa were identified; of those, the genera *Rhodococcus*, *Skermania*, *Aldersonia*, *Streptomyces*, and Mycobacterium were the most frequent donor taxa (Fig. 7A). The number of HGT genes in tested isolates ranged from 3,717 to 4,099 (Fig. 7B). Interestingly, compared with clade D, clade A and B isolates have more HGT-acquired genes, suggesting that these have undergone a higher degree of HGT events (*P* < 0.001, one-way analysis of variance [ANOVA]) (see Fig. S3 in the supplemental material).

10.1128/msystems.01406-21.2 FIG S2 Distribution of functional categories for the *N. cyriacigeorgica* complex. (A) Functional categories for pan-genome, core orthogroups, accessory orthogroups, horizontally transferred genes, prophage genes, and plasmid genes. (B) Number of orthogroups in each gene set. Download FIG S2, PDF file, 0.1 MB. Copyright © 2022 Xu et al. 2022 Xu et al. https://creativecommons.org/licenses/by/4.0/ This content is distributed under the terms of the Creative Commons Attribution 4.0 International license.

10.1128/msystems.01406-21.3 FIG S3 Number of horizontally transferred genes per clade of the *N. cyriacigeorgica* complex. Download FIG S3, PDF file, 0.1 MB. Copyright © 2022 Xu et al. 2022 Xu et al. https://creativecommons.org/licenses/by/4.0/ This content is distributed under the terms of the Creative Commons Attribution 4.0 International license.

Mobile genetic elements (MGEs) largely impact genome structure and function, facilitating HGT and adaptive evolution (28). Thus, we next investigated the distribution of MGEs, including insertion sequence (IS), prophage, and plasmids. A diversity of IS elements was identified, including 15 IS families (see Fig. S4 in the supplemental material). Members of the IS*3* families were the most common. A total of 203 prophage regions marked as questionable, incomplete, or intact were found in the genomes analyzed (Fig. 7C), and the average length of the prophage sequence was ∼17.2 kb. Two strains (DSM 43004 and DSM 43005) carried the highest number of prophage fragments (7 prophages). Ten strains carried the intact prophages. These 203 prophages represented 59 prophage types (see Fig. S5 in the supplemental material). No robust correlation was established between prophage type and clades. A total of 825 gene families were associated with prophages in the pan-genome and mainly enriched the category of “S: function unknown” (651 genes, 78.9%), followed by “L: replication, recombination, and repair” (46,5.6%) and “K: transcription” (42, 5.1%) (Fig. S2). Plasmid sequences were detected in 92.3% of the test genomes (Fig. 7C). Further analysis showed that genes on plasmids were shared among chromosomes. Specifically, we observed 7,363 gene families carried on plasmids; of those, 6,654 were also on chromosomes, suggesting frequent recombination between strains. Annotation of these plasmid sequences showed that 168 of 507 virulence genes and 58 of 125 antibiotic resistance genes were located in plasmids.

10.1128/msystems.01406-21.4 FIG S4 Heatmap of the distribution of IS elements of the *N. cyriacigeorgica* complex. Download FIG S4, PDF file, 0.2 MB. Copyright © 2022 Xu et al. 2022 Xu et al. https://creativecommons.org/licenses/by/4.0/ This content is distributed under the terms of the Creative Commons Attribution 4.0 International license.

10.1128/msystems.01406-21.5 FIG S5 Prophage profile of the *N. cyriacigeorgica* complex. Presence is indicated by blue cells and absence by white cells. Download FIG S5, PDF file, 0.3 MB. Copyright © 2022 Xu et al. 2022 Xu et al. https://creativecommons.org/licenses/by/4.0/ This content is distributed under the terms of the Creative Commons Attribution 4.0 International license.

### Virulence gene profiles reflect clade-specific pathogenic differences.

To provide a comprehensive view of NCC virulence, we analyzed the virulence gene profile of 91 isolates by BLASTp against the Virulence Factors Database (VFDB). We found 507 virulence-related genes in the pan-genome of NCC, and on average, each genome contained about 295 virulence factors. Approximately 46.3% of the virulence genes were potentially acquired by HGT, 161 of which were mediated by MGEs. Furthermore, 129 virulence factors were encoded by core genes, including adherence, invasion, toxin, type VII secretion system (T7SS), stress adaptation, anaerobic respiration, iron uptake, antiphagocytosis, and mycolic acid synthesis, suggesting common pathogenic mechanisms of NCC (see Table S4 in the supplemental material).

10.1128/msystems.01406-21.9 TABLE S4 List of predicted virulence factors in the core genome. Download Table S4, PDF file, 0.09 MB. Copyright © 2022 Xu et al. 2022 Xu et al. https://creativecommons.org/licenses/by/4.0/ This content is distributed under the terms of the Creative Commons Attribution 4.0 International license.

**FIG 8:**
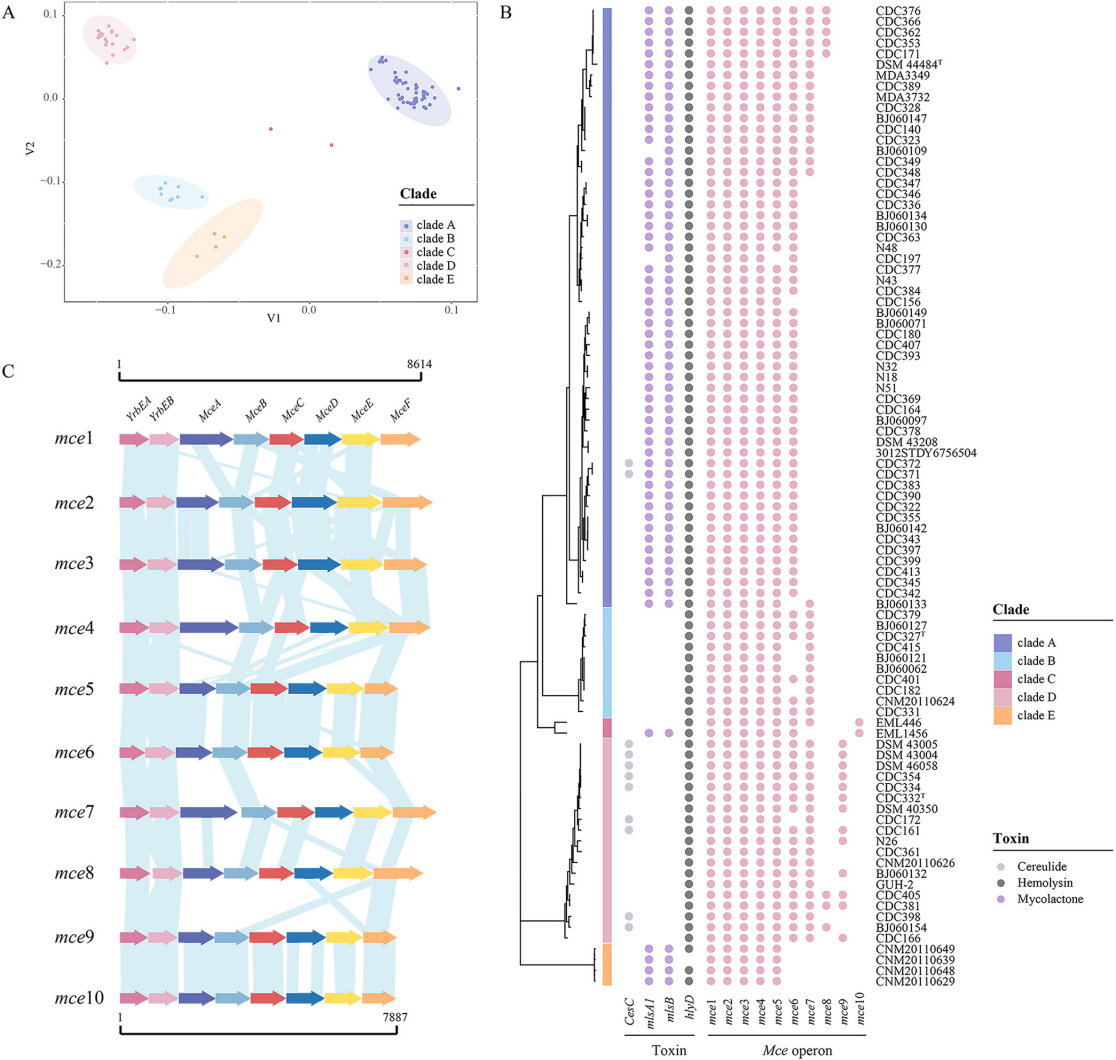
Virulence genes in the genomes of *N. cyriacigeorgica* complex. (A) Differences among virulence genes per clade by PCoA plot. (B) Distribution of the toxin-coding genes and *mce* operons. Each dot indicates the presence of the gene or operon. (C) Comparison of different types of *mce* operons. Regions of homology are indicated by blue shading.

A PCoA plot revealed five distinct clusters of genomes, implying species-specific differences in pathogenicity (Fig. 8A). We consequently further analyzed clade-specific virulence factors to explore the differences in pathogenicity among the members of the NCC. Although *hlyD*-encoded hemolysin was present in almost all strains, three genes encoding toxins showed different distributions among species (Fig. 8B). The mycolactone-coding genes *mlsA1* and *mlsB* were shared by clades A and E and one strain (EML1456) in clade C and were absent from clades B and D. Mycolactone is a macrolide toxin that can cause cell death via apoptosis (29, 30). The presence of these two genes indicates that these strains may have cytotoxic and immunosuppressive activity. The gene *cesC* encodes the mitochondriotoxic depsipeptide cereulide, leading to clinical complications such as hepatotoxicity, encephalopathy, and β-cell dysfunction, present in nine strains in clade D and two strains in clade A (31, 32).

The ability to invade host cells has a pivotal role in establishing bacterial infection. *mce* family genes, which are essential for the entry of *Nocardia* into the mammalian cell and their survival within phagocytes and epithelial cells (33, 34), were detected widely in tested NCC isolates. The pan-genome of NCC contained 10 types of gene clusters, including the *mce* operon. In general, the overall gene arrangement was similar in the NCC, but the diversity of the genes in the *mce* operon was correlated with the clade that they originated from (Fig. 8C). For example, the *mce6* operon was present in all clades except clade E. The *mce9* and *mce10* operons were present exclusively in clades C and D, respectively, suggesting that they contributed to a selective survival advantage for these two clades.

### Clade-related differences of antibiotic resistance genes (ARGs) and phenotypic profiles in NCC.

**FIG 9:**
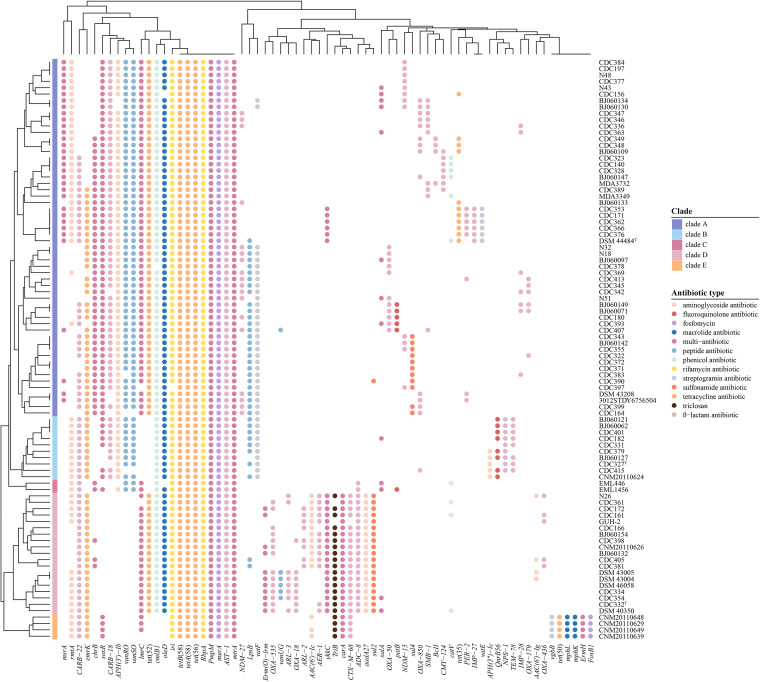
Distribution pattern of ARGs in the genomes of *N. cyriacigeorgica* complex. Each dot indicates the presence of a gene. Different colors indicate different types of ARGs.

In this study, 125 ARGs in the pan-genome were identified by BLASTp against the Comprehensive Antibiotic Resistance Database (CARD). These ARGs are involved at least 12 antimicrobial classes of drugs: ranging from β-lactam antibiotics to sulfonamides (Fig. 9). Nine ARGs in the NCC core genome were found to be resistant to tetracycline [*tet*(56), *tetA*(58), *tetB*(58)], rifamycin (*iri*, *rbpA*), β-lactams (*bla*_AST-1_), fluoroquinolone (*pmpM*), fosfomycin (*murA*), and macrolide (*mtrA*). In addition, 11 unique genes, including *adc-73*, *arr*-*5*, *edeQ*, *erm*(41), *fosC*2, *tet*(33), *golS*, *hmrM*, *rlmA*(II), *mphA*, and *lra-17*, were inferred to be introduced by plasmid-mediated HGT, which may have helped in adapting to environmental changes. Remarkably, different NCC clades have different ARG distribution patterns: for example, except for clade E, clades A to D carried *cmlB1* and *oleD*, while only clade E carried *vrgB*, *tet*(50), *mphL*, *mphK*, *ermH*, and *fosB1*. The *vanRO* and *vanSO* genes were present in clades A to C but absent in clades D and E.

A total of 60 NCC isolates, including 39 in clade A, 6 in clade B, and 15 in clade D, were tested for antibiotic susceptibility against 19 antibiotics (Table 1). Although related ARGs were identified, all isolates were susceptible to tobramycin, amikacin, doxycycline, minocycline, tigecycline, trimethoprim-sulfamethoxazole, and linezolid. Almost all isolates exhibited resistance to fluoroquinolone antibiotics, with 96% showing resistance to ciprofloxacin and levofloxacin, followed by moxifloxacin (75%). The majority of isolates (>85%) displayed resistance to β-lactam antibiotics (cefoxitin, amoxicillin-clavulanic acid, imipenem), kanamycin, and clarithromycin. The highest MIC_50_ and MIC_90_ values against NCC were observed for vancomycin and streptomycin. The gene cluster including *vanSO* and *vanRO* was identified in clade A and B isolates, conferring vancomycin resistance. However, vancomycin resistance (10/15 [66%]) among the clade D isolates without this gene cluster was observed, indicating the existence of other resistance mechanisms. Interestingly, a correlation between phylogenetic clade and streptomycin resistance was observed. Except for isolate CDC415, the isolates in clades A and B carried the *vatF* gene related to streptomycin resistance, and these strains exhibited higher streptomycin resistance rates than isolates in clade D.

**TABLE 1:** Antimicrobial resistance among clades and correlations with antibiotic resistance gene presence

Drug type	Drug^*a*^	Concn range (μg/mL)	No. (%) of resistant isolates	Clades^*b*^	Predicted ARGs^*b*^	MIC (μg/mL)^*c*^		
						Range	MIC_50_	MIC_90_
β-Lactam antibiotics	AXO	4 to 64	6 (10)	A (5), B (0), D (1)	*bla*_NDM-27_ (12), *bla*_NDM-15_ (7), *bla*_IMP-28_ (5), *bla*_IMP-27_ (6), *bla*_OXA-50_ (5), *bla*_SMB-1_ (6), *bla*_OXA-179_ (4), and *bla*_OXA-436_ (4) are only in clade A; *bla*_OXA-535_ (11) and *bla*_OXA-18_ (8) are only in clade D; *bla*_OXA-850_ (9)	<4 to >64	<4	>64
	FOX	4 to 128	57 (95)	A (38), B (6), D (13)		64 to >128	>128	>128
	AUG2	1 to 64	54 (90)	A (37), B (6), D (11)		16/8 to >64/32	64/32	>64/32
	FEP	1 to 32	30 (50)	A (22), B (1), D (7)		4 to >32	32	>32
	IMI	2 to 64	52 (86)	A (35), B (6), D (11)		<2 to >64	32	>64

Aminoglycoside antibiotics	KAN	0.5 to 256	54 (90)	A (36), B (5), D (13)	*aadA27* (15) and *aac(6′)*-*Ig* (3) are only in clade D; *aph(3′)-Ib* (47); *rmtA* (39); *aac(6′)*-*Ic* (8); *aph(3′′)*-*Ic* (5); *ykkC* (21)	16 to 256	64	256
	TOB	1 to 16	0 (0)	0		<1	<1	<1
	AMI	1 to 64	0 (0)	0		<1	<1	<1

Streptogramin antibiotic	STR	0.5 to 256	38 (63)	A (34), B (4), D (0)	*vatF* (25) is in clades A and B; *vatE* (6) is only in clade A	1 to >256	>256	>256

Fluoroquinolone antibiotics	MXF	0.25 to 8	45 (75)	A (31), B (2), D (12)	*pmpM* (60) is a core gene; *soxR* (43) is in clades A and B; *qnrB*56 (4) is only in clade B; *patB* (3) is only in clade A	2 to >8	4	8
	CIP	0.12 to 4	58 (96)	A (37), B (6), D (15)		2 to >4	>4	>4
	LVX	0.5 to 256	58 (96)	A (37), B (6), D (15)		2 to 32	8	32

Glycopeptide antibiotic	VAN	0.5 to 256	55 (81)	A (34), B (5), D (10)	*vanSO*-*vanRO* gene cluster (42) is in clades A and B; *vanUG* (6)	0.5 to >256	256	>256

Macrolide antibiotic	CLA	0.06 to 16	52 (86)	A (35), B (6), D (11)	*mtrA* is a core gene; *oleD* (59); *salA* (6); *chrB* (34) and *msrA* (23) are only in clade A; *erm*(O)-*lrm* (9) and *carA* (15) are only in clade D; *lmrC* (52) is in clades A and D	2 to >16	>16	>16

Tetracycline antibiotics	DOX	0.12 to 16	0 (0)	0	*tet*(56), *tetA*(58), and *tetB*(58) are core genes; *tet*(52) (90); *emrK*(45); *tet*(35) (9)	0.25 to 4	2	4
	MIN	1 to 8	0 (0)	0		<1 to 4	2	4
	TGC	0.015 to 4	0 (0)	0		0.25 to 2	1	1

Sulfonamide antibiotic	SXT	0.25 to 152	0 (0)	0	*sul2* (16); *sul4* (10) is only in clade A	<0.25/4.75 to 0.5/9.5	<0.25/4.75	0.5/9.5

Oxazolidinone antibiotic	LZD	1 to 32	0 (0)	0	*lmrC* (52) is in clades A and D; *msrA* (23) is only in clade A; *carA* (15) is only in clade D; *salA* (6)	<1 to 2	<1	2

a AXO, ceftriaxone; FOX, cefoxitin; AUG2, amoxicillin-clavulanic acid; FEP, cefepime; IMI, imipenem; KAN, kanamycin; TOB, tobramycin; AMI, amikacin; STR, streptomycin; MXF, moxifloxacin; CIP, ciprofloxacin; LVX, levofloxacin; VAN, vancomycin; CLA, clarithromycin; DOX, doxycycline; MIN, minocycline; TGC, tigecycline; SXT, trimethoprim-sulfamethoxazole; LZD, linezolid.

b Values in parentheses show the number of isolates resistant to a drug.

c The MIC is defined as the lowest concentration of a drug that completely inhibits the visible growth of the tested isolates, the MIC_50_ is defined as the MIC value of a drug capable of inhibiting the growth of 50% of tested isolates, and the MIC_90_ is defined as the MIC value of a drug capable of inhibiting the growth of 90% of tested isolates.

## DISCUSSION

To outline the population structure, genetic diversity, evolutionary dynamics, and pathogenic traits of the species *N. cyriacigeorgica*, we performed comparative genomics using 91 whole-genome sequences from clinical and environmental sources. We found that *N. cyriacigeorgica* is not a monotypic species but is composed of five species-level clades (A to E), namely, the *N. cyriacigeorgica* complex (NCC), based on the fast-evolving *dapb1* gene, concatenated 2,935 single-copy core genes’ phylogenies, and ANI analysis. However, the 16S rRNA gene failed to discriminate strains into their respective clades, indicating that it strongly underestimated diversity in the NCC.

Three dominant clades (A, B, and D) contained all the clinical isolates, indicating their potential to colonize and cause infections in humans. The most frequently recovered species was *N. cyriacigeorgica* (clade A), with 56 isolates, harboring 68.8% (53/77) of clinical isolates and found to have the most varied geographic range. Several reports have shown that some *Nocardia* species are more prevalent in specific regions due to the influence of climate, vegetation type, and soil pH (2, 35, 36). In our study, particular species seemed to be associated with a regional restriction, such as environmental clades C and E, which were exclusively found in France and Venezuela, respectively. Given that the isolate sources were from only five countries with a bias toward China, this inference requires verification as more genomes from origins worldwide become available. Future large-scale studies on the epidemiology of NCC are needed to understand the geographic structure of NCC per clade.

The PCoA demonstrated that the five clades differed substantially based on divergent orthogroup content. Moreover, the degree of intraclade heterogeneity varied, and clade A showed a much higher species-level diversity relative to the other clades. For instance, strain BJ060133 was segregated from other members of clade A in the PCoA plot. The concatenated single-copy core genes’ phylogeny (three subclades) and pairwise ANI values (∼5% ANI distance, slightly lower than the threshold for species delimitation) also support this observation. This investigation prompts a need to further explore intraspecies variation in clade A.

The observed distinction in orthogroup composition raised the question of whether there were differences in the general functional profiles among clades. To answer this question, we mapped all orthogroups against the eggNOG database and then conducted a PCoA based on 18 functional categories of orthogroup content. The differential orthogroup subsets were involved in nine categories, indicating significant differences in the functional capacities of the five clades. Consistent with previous studies (37–39), a positive correlation was observed between species-level taxa and functional divergence. A considerable number of genes with unexplored roles are nevertheless present in these understudied populations, thereby limiting understanding of their contributions to function. These observations highlight the need for further *in vitro* experiments to characterize the phenotypic characteristics of the five NCC clades.

Next, we examined how natural selection influences genetic diversity across the NCC. Although the vast majority of single-copy core genes in NCC were strongly constrained by purifying selection, we observed *K_a_*/*K_s_* ratio variations in interclades: the higher *K_a_*/*K_s_* ratios in clades D and E than in other clades. These increases in *K_a_*/*K_s_* ratios may be driven by ecological dynamics or shifts in environmental conditions. Furthermore, we identified two signatures of positive selection in clades D and E. There are many well-documented examples in which strong positive selection has influenced rapid bacterial adaptation (40–42). In some cases, adaptations of menaced bacterial populations to a new niche can occur by frequent mutations. It can therefore be inferred that the two clade-specific mutations may have led to adaptations to survive in stressful environments. The two signatures of positive selection might have played a pivotal role in the evolutionary dynamics of NCC. However, the function of these two gene families remains poorly characterized and requires further investigation.

We further explored HGT events in the NCC and compared their characteristics. Although HGT events were evident in the NCC isolates, they appeared to be different in extent and to have occurred at the highest frequency in clade A. Given that HGT is responsible for bacterial speciation and subspeciation, one question arises from this observation (26, 27). Specifically, it is unclear if HGT leads to the higher evolutionary divergence of clade A compared to the other clades while also contributing to the emergence of new subspecies. However, this question is not easy to answer because of evolutionary complexity. First, it is difficult to discriminate between the orthogroups that affect speciation events. Such clades would be considered different species in some orthogroups but the same clade in other orthogroups. Second, point mutations that modify existing genes have also contributed to diversification. Thus, additional information is needed to distinguish between convergent evolution and HGT. Nevertheless, the results described above are insufficient to assess the role of HGT events in the NCC speciation.

Finally, we profiled the potential pathogenic characteristics of NCC strains. The environmental clades (C and E) were equipped with some of the same virulence genes as human pathogen clades (A, B, and D), such as putative hemolysis genes (*hlyD*). A prior study using a murine model showed that the environmental isolate EML446, which is a member of clade C, has significant infectivity, with pulmonary damage similar to that of clinical isolate GUH-2 in clade D (19). These data highlight that environmental isolates could be capable of causing disease and have the potential to transition from an environmental niche and emerge as pathogens.

PCoA revealed that the virulence profiles of five clades were distinct, suggesting distinct genetic strategies for host adaptation in the NCC clades. Previous studies have shown virulence heterogeneity, like the higher virulence of clade A strains DSM 43208 and DSM 44484^T^ compared to clade D strain GUH-2 in challenged mice (43, 44). A possible explanation for this might be the presence of additional toxin-coding genes (*mlsA1* and *mlsB*) in clade A, and this hypothesis needs to be further explored. In addition, the environmental clade (E) has only been characterized by Carrasco et al., and its pathogenicity remains unclear (36). To develop a comprehensive understanding of NCC pathogenicity traits, additional studies are needed to evaluate and compare virulence traits among the five clades *in vitro* and *in vivo*.

In conclusion, our study is the first, to our knowledge, to perform a systematic comparative genomic analysis of the NCC, allowing us to obtain a comprehensive picture of the population diversity, evolution, and pathogenicity of this underinvestigated pathogen. Our study revealed that the NCC comprises five distinct species-level clades that vary in functional features, selection pressure, pathogenicity, and antibiotic resistance at the genomic level. All five clades exhibited the potential for pathogenesis, particularly *N. cyriacigeorgica* (clade A), strains of which may potentially evolve into highly adapted human pathogens. Collectively, these results suggest that NCC is far more diverse than previously assumed. However, several questions remain unresolved, and further research is needed to explore the functions of the candidate genes identified here.

## MATERIALS AND METHODS

### Bacterial strains and culture conditions.

Forty-five clinical isolates of *N. cyriacigeorgica* were collected from 12 provinces in China between 2010 and 2019 (Table S1). Stock cultures in brain heart infusion broth (BHI) mixed with 25% glycerol were stored at −80°C. Bacterial cultures were performed using BHI agar plates with 5% sheep blood and incubated at 37°C with agitation for 48 to 72 h.

### Genome sequencing, assembly, and annotation.

According to the manufacturer’s instructions, the genomic DNA of 45 *N. cyriacigeorgica* strains was extracted using the Wizard genomic DNA purification kit (Promega, Madison, WI, USA), with an extra cell lysis step using 50 mg/mL of lysozyme. Whole-genome sequencing was performed using the Illumina NovaSeq platform in the PE150 mode to generate 350-bp paired-end-read libraries using NEBNext DNA library prep kit (New England Biolabs, USA), giving all sequencing depths exceeding 100-fold. The raw sequence data were filtered to remove reads containing >40 bp of low-quality bases, >10 bp of N bases, or overlaps between reads and adapters of >15 bp by using the software readfq v10. After filtering, high-quality reads were *de novo* assembled into contigs using SPAdes v3.8.0 (45). The CDSs were predicted by Prokka v1.13 (46).

### Downloading of publicly available assemblies and quality control.

All genome sequences annotated as *N. cyriacigeorgica* were downloaded from the National Center for Biotechnology Information (NCBI) and GSA on 29 May 2021, using in-house-scripts (Table S1). All genome assemblies were subjected to quality control by Quast v5.0.2 and CheckM v1.1.3 (47, 48), with the criteria of *N*_75_ values of >10,000 bp, <500 undetermined bases per 100,000 bases, and a completeness of >98% (37). One genome, BJ06-0118 (GCA_015478155.1), was removed. Finally, 46 publicly available genomes and 45 newly sequenced assemblies were subjected to further analysis, resulting in a pool of 91 genomes of *N. cyriacigeorgica*.

### Analysis of 16S rRNA sequences, genome size, and GC content.

The 16S rRNA sequences were extracted from all genomes. Each sequence was aligned to the 16S rRNA sequence of *N. cyriacigeorgica* strain DSM 44484^T^ by using BLASTn with default parameters. The genome size, number of CDSs, and GC content were estimated using Quast v5.0.2 and CheckM v1.1.3.

### Phylogenetic tree construction.

To infer phylogenetic relationships within *N. cyriacigeorgica*, we performed a phylogenetic analysis following the previously described method (49). Briefly, a nonredundant homologous gene set of *N. cyriacigeorgica* DSM 44484^T^ was computed using CD-HIT v4.6.6 (50). To identify homologous genes, this gene set was aligned to the CDSs of all genomes by using BLASTn, with an E value threshold of 1e−10 and a coverage of 60%. Single-copy core genes were identified when the homologous gene was just one copy and present in over 96% of genomes. This method produced a single-copy core gene set of 2,935 genes. Each single-copy core gene sequence was aligned separately using ClustalW2 and then merged. We then constructed an ML tree based on the final alignment by using the software iqtree v1.6.11 and the GTR+I+G model with 1,000 bootstrap replicates, rooted by the genome sequence of *N. carnea* DSM 43397^T^ (GCA_000308515.1) (51). The resulting phylogenetic tree was visualized using the R package ggtree v2.2.4 (52), and bootstrap values were indicated at each node. The *dapb1* gene sequences were extracted and aligned and then used to construct a phylogeny as described above.

### Calculation of average nucleotide identity.

Pairwise ANI values across all genomes were calculated using a Perl script as described by Li et al. (49). A matrix with ANI values across all genomes was visualized using the R package pheatmap.

### Phenotypic characterization of representative strains.

Growth conditions were performed by assessing their growth at 4°C, 15°C, 28°C, 30°C, 37°C, 40°C, and 50°C in liquid BHI without shaking under anaerobic conditions. Salt tolerance (1.5 to 5.5% [wt/vol] NaCl; 1% intervals) and different pHs (range, 3.0 to 11.0; 1% intervals) were also tested in BHI broth at 37°C. The isolates were characterized biochemically using API 50 CH strips (for carbohydrate fermentation) and API ZYM (for enzymatic profiling). The cellular fatty acids were extracted according to the Sherlock Microbial Identification System protocols and identified using an Agilent 6890N gas chromatograph (Agilent Technologies, Santa Clara, CA, USA).

Type strains of new species (CDC327^T^ and CDC332^T^) were deposited in the Japan Collection of Microorganisms (JCM) and the Guangdong Microbial Culture Collection Center (GDMCC).

### Pan-genome analysis and functional annotation.

The software OrthoFinder v2.4.0 was used to identify orthogroups among all tested genomes, with default parameters (24). The genes present in >96% of the genomes analyzed were defined as core orthogroups, and the other orthogroups were defined as accessory orthogroups. All orthogroups were annotated using emapper v2.0.1 against the eggNOG v5.0 database (53). The PCoA based on orthogroup profiles was performed using the R package vegan.

### Selective pressure analysis.

Evolutionary pressure analyses of single-copy core genes were conducted by calculating *K_a_*/*K_s_* ratios using the KaKs_Calculator v2.0 (54). Differences in *K_a_*/*K_s_* ratios between distinct clades were assessed using Wilcoxon’s two-sided test. Differences with a *P* value of <0.05 were considered statistically significant.

### Identification of potential HGTs and MGEs.

HGTs were predicted by the software HGTector v2.0, using the cutoffs of 60% identity, 60% coverage, and an E value of 1e−6 (55). Differences in the total number of HGTs among distinct clades were assessed using one-way ANOVA. Statistical significance was determined using a cutoff *P* value of <0.05. IS elements were predicted using the ISEScan v1.7.2.2 with default criteria (56). The PHASTER (PHAge Search Tool Enhanced Release) was used to identify the prophage sequences within genome assemblies (57). Predictions of plasmid sequences were performed using PlasFlow v1.1 (58).

### Identification of virulence factors and antibiotic resistance genes.

To identify the virulence factors, the protein sequences of all genomes were aligned using BLASTp with cutoffs of 40% identity, 70% coverage, and an E value of 1e−10 against the data set from the VFDB (59). To predict the *mce* operon, hidden Markov models (HMMs) of Mce proteins (PF02470) and YrbE proteins (PF02405) were downloaded from the Pfam database, and then HMMER was used to scan the pan-genome against these two HMMs (60). The *mce* operon was defined as having at least four orthogroups present and close to one another in the genome. The ARGs were predicted using Resistance Gene Identifier (RGI) v5.1.1 with cutoffs of 60% identity and 70% coverage against the data set from the CARD (61).

### *In vitro* antibiotic susceptibility test.

A total of 60 NCC isolates, including 45 from this study and 15 previously described isolates, were tested for antimicrobial susceptibility (21). The MICs of ceftriaxone (AXO), cefoxitin (FOX), amoxicillin-clavulanic acid (AUG2), cefepime (FEP), imipenem (IMI), tobramycin (TOB), amikacin (AMI), moxifloxacin (MXF), ciprofloxacin (CIP), levofloxacin (LVX), clarithromycin (CLA), doxycycline (DOX), minocycline (MIN), tigecycline (TGC), trimethoprim-sulfamethoxazole (SXT), and linezolid (LZD) were determined using commercial broth microdilution panels (TREK Sensititre Rapmyco microdilution panel; Thermo Fisher Scientific, Inc., Sunnyvale, CA, USA), following the manufacturer’s instructions. The antimicrobial susceptibility testing of kanamycin (KAN), streptomycin (STR), and vancomycin (VAN) was performed by broth microdilution according to standard protocols. The drug dilution ranges tested for isolates are listed in Table 1, and resistance was defined using the Clinical and Laboratory Standards Institute (CLSI) criteria. All tests for each strain were conducted in duplicate on different days. Escherichia coli ATCC 35218, Staphylococcus aureus ATCC 25923, Staphylococcus aureus ATCC 29213, and Pseudomonas aeruginosa ATCC 27853 were used as quality control strains.

### Data availability.

The raw sequence data of the 45 newly sequenced isolates are available under accession no. CRA005399 within the GSA (https://bigd.big.ac.cn/gsa/) (62).
