# Supplementary material for: Comprehensive Analysis of the Nocardia cyriacigeorgica Complex Reveals Five Species-Level Clades with Different Evolutionary and Pathogenicity Characteristics
Source: mSystems. 2022 Apr 18;7(3):e01406-21. doi: 10.1128/msystems.01406-21 (PMC9239197; doi:10.1128/msystems.01406-21)
Supplement: TABLE S1 [file msystems.01406-21-s0006.pdf]

Table S1. Genome sequences of strains used in this study.

Genome	Isolated source	Year	Origin	Genome Size (Mb)	No. of CDSs	%GC	No. of scaffolds	Accession Number	Clade	New species proposition	References
3012STDY6756504	NA	NA	NA	6.48	5787	68.24	1	GCA_900683635.1	A	*N. cyriacigeorgica*	
BJ060062	sputum	2019	China: Beijing	6.33	5694	68.43	7	GCA_015478435.1	B	*N. ningxiensis*	
BJ060071	Pus	2018	China: Guangxi	6.45	5811	68.21	20	GCA_015478465.1	A	*N. cyriacigeorgica*	
BJ060097	BALF	2019	China: Changsha	6.71	6198	68.22	98	GCA_015478375.1	A	*N. cyriacigeorgica*	
BJ060109	sputum	2018	China: Guangxi	5.92	5342	68.46	25	GCA_015478255.1	A	*N. cyriacigeorgica*	
BJ060121	sputum	2019	China: Beijing	6.33	5729	68.42	80	GCA_015478165.1	B	*N. ningxiensis*	
BJ060127	sputum	2019	China: Beijing	6.37	5713	68.38	8	GCA_015478075.1	B	*N. ningxiensis*	
BJ060130	sputum	2020	China: Beijing	6.43	5794	68.23	15	GCA_015478055.1	A	*N. cyriacigeorgica*	
BJ060132	sputum	2019	China: Beijing	6.38	5835	68.3	61	GCA_015477975.1	D	*N. yinchuanensis*	
BJ060133	sputum	2019	China: Beijing	6.14	5586	68.36	44	GCA_015477995.1	A	*N. cyriacigeorgica*	
BJ060134	sputum	2019	China: Beijing	6.43	5794	68.23	18	GCA_015477945.1	A	*N. cyriacigeorgica*	
BJ060142	sputum	2019	China: Beijing	6.32	5671	68.31	31	GCA_015477865.1	A	*N. cyriacigeorgica*	
BJ060147	sputum	2019	China: Beijing	6.69	6192	68.23	39	GCA_015477705.1	A	*N. cyriacigeorgica*	
BJ060149	sputum	2019	China: Zhengzhou	6.57	5923	68.19	34	GCA_015477725.1	A	*N. cyriacigeorgica*	
BJ060154	BALF	2019	China: Beijing	6.26	5868	68.35	202	GCA_015477645.1	D	*N. yinchuanensis*	
DSM 40350	septicemic actinomycosis of dog	1975	NA	6.10	5551	68.48	21	CRX204787	D	*N. yinchuanensis*	(1)
DSM 43004	NA	1990	NA	6.54	6180	68.38	24	CRX204786	D	*N. yinchuanensis*	(1)

DSM 43005	NA	NA	NA	6.54	6176	68.38	25	CRX204785	D	*N. yinchuanensis*	(1)
DSM 43208	pus from empysema	1990	NA	6.65	5960	68.26	31	CRX204784	A	*N. cyriacigeorgica*	(1)
DSM 46058	NA	1979	NA	6.36	5903	68.4	54	CRX204783	D	*N. yinchuanensis*	(1)
CDC140	sputum	2015	China: Beijing	6.20	5595	68.38	12	CRX204782	A	*N. cyriacigeorgica*	(1)
CDC156	sputum	2015	China: Beijing	6.68	6090	68.29	34	CRX204776	A	*N. cyriacigeorgica*	(1)
CDC161	sputum	2015	China: Beijing	6.22	5692	68.45	50	This study	D	*N. yinchuanensis*	
CDC164	sputum	2015	China: Beijing	6.45	5810	68.28	34	This study	A	*N. cyriacigeorgica*	
CDC166	sputum	2015	China: Beijing	6.16	5648	68.42	113	This study	D	*N. yinchuanensis*	
CDC171	sputum	2015	China: Beijing	6.36	5759	68.19	42	This study	A	*N. cyriacigeorgica*	
CDC172	sputum	2015	China: Beijing	6.10	5585	68.47	48	This study	D	*N. yinchuanensis*	
CDC180	sputum	2015	China	6.48	5827	68.23	40	This study	A	*N. cyriacigeorgica*	
CDC182	sputum	2015	China: Neimenggu	6.48	5840	68.38	11	CRX204768	B	*N. ningxiensis*	(1)
CDC197	lung	2016	China: Zhejiang	6.49	5885	68.35	12	CRX204760	A	*N. cyriacigeorgica*	(1)
CDC322	pus	2016	China: Ningxia	6.54	5888	68.26	28	CRX204752	A	*N. cyriacigeorgica*	(1)
CDC323	special material	2016	China: Ningxia	6.42	5848	68.37	19	CRX204751	A	*N. cyriacigeorgica*	(1)
CDC327^T	sputum	2016	China: Ningxia	6.23	5631	68.46	19	CRX204748	B	*N. ningxiensis*	(1)
CDC328	sputum	2016	China: Ningxia	6.18	5565	68.39	41	This study	A	*N. cyriacigeorgica*	
CDC331	sputum	2016	China: Ningxia	6.27	5640	68.46	26	This study	B	*N. ningxiensis*	
CDC332^T	alveolar lavage fluid	2016	China: Ningxia	6.21	5666	68.46	49	CRX204747	D	*N. yinchuanensis*	(1)
CDC334	sputum	2016	China: Ningxia	6.32	5912	68.47	54	This study	D	*N. yinchuanensis*	
CDC336	sputum	2016	China:	6.52	5953	68.16	31	This study	A	*N. cyriacigeorgica*	

			Ningxia							
CDC342	surgical drain	2019	China: Shandong	6.51	5874	68.26	48	This study	A	*N. cyriacigeorgica*
CDC343	sputum	2010	China	6.48	5837	68.28	36	This study	A	*N. cyriacigeorgica*
CDC345	sputum	2013	China: Beijing	6.45	5861	68.32	41	This study	A	*N. cyriacigeorgica*
CDC346	pus	2014	China: Hubei	6.30	5655	68.27	31	This study	A	*N. cyriacigeorgica*
CDC347	sputum	2014	China: Hubei	6.30	5654	68.27	36	This study	A	*N. cyriacigeorgica*
CDC348	BALF	2014	China: Anhui	6.31	5694	68.36	34	This study	A	*N. cyriacigeorgica*
CDC349	sputum	2014	China: Anhui	6.31	5698	68.36	38	This study	A	*N. cyriacigeorgica*
CDC353	BALF	2015	China: Anhui	6.29	5672	68.2	43	This study	A	*N. cyriacigeorgica*
CDC354	sputum	2015	China: Hebei	6.25	5713	68.43	37	This study	D	*N. yinchuanensis*
CDC355	BALF	2015	China: Neimenggu	6.27	5609	68.32	44	This study	A	*N. cyriacigeorgica*
CDC361	sputum	2016	China	6.22	5686	68.42	36	This study	D	*N. yinchuanensis*
CDC362	sputum	2016	China: Beijing	6.44	5844	68.19	53	This study	A	*N. cyriacigeorgica*
CDC363	sputum	2016	China: Shanxi	6.20	5573	68.3	39	This study	A	*N. cyriacigeorgica*
CDC366	sputum	2016	China	6.38	5773	68.19	79	This study	A	*N. cyriacigeorgica*
CDC369	BALF	2016	China	6.55	5922	68.19	33	This study	A	*N. cyriacigeorgica*
CDC371	sputum	2017	China: Hebei	6.37	5752	68.32	67	This study	A	*N. cyriacigeorgica*
CDC372	BALF	2017	China: Jiangsu	6.49	5866	68.31	54	This study	A	*N. cyriacigeorgica*
CDC376	BALF	2017	China: Beijing	6.47	5869	68.19	53	This study	A	*N. cyriacigeorgica*
CDC377	sputum	2017	China: Beijing	6.17	5621	68.35	40	This study	A	*N. cyriacigeorgica*
CDC378	sputum	2017	China: Beijing	6.53	5883	68.21	38	This study	A	*N. cyriacigeorgica*

CDC379	sputum	2017	China: Hebei	6.35	5706	68.39	35	This study	B	*N. ningxiensis*	
CDC381	sputum	2018	China: Beijing	6.18	5673	68.45	34	This study	D	*N. yinchuanensis*	
CDC383	sputum	2018	China: Beijing	6.21	5585	68.37	48	This study	A	*N. cyriacigeorgica*	
CDC384	sputum	2018	China: Henan	6.38	5814	68.27	43	This study	A	*N. cyriacigeorgica*	
CDC389	sputum	2018	China: Shanxi	6.30	5713	68.38	63	This study	A	*N. cyriacigeorgica*	
CDC390	sputum	2018	China: Beijing	6.50	5881	68.27	64	This study	A	*N. cyriacigeorgica*	
CDC393	sputum	2019	China: Zhejiang	6.76	6105	68.14	67	This study	A	*N. cyriacigeorgica*	
CDC397	sputum	2019	China: Beijing	6.25	5616	68.3	30	This study	A	*N. cyriacigeorgica*	
CDC398	sputum	2019	China	6.29	5794	68.36	127	This study	D	*N. yinchuanensis*	
CDC399	sputum	2019	China	6.50	5921	68.29	38	This study	A	*N. cyriacigeorgica*	
CDC401	sputum	2019	China: Hebei	6.46	5861	68.37	27	This study	B	*N. ningxiensis*	
CDC405	BALF	2019	China: Beijing	6.46	5872	67.73	67	This study	D	*N. yinchuanensis*	
CDC407	sputum	2019	China: Beijing	6.57	5976	68.19	31	This study	A	*N. cyriacigeorgica*	
CDC413	sputum	2019	China: Beijing	6.41	5797	68.31	40	This study	A	*N. cyriacigeorgica*	
CDC415	sputum	2019	China: Beijing	6.31	5709	68.34	67	This study	B	*N. ningxiensis*	
CNM20110624	soil	2002	Venezuela: Lara	6.34	6148	68.41	461	GCA_010858045.1	B	*N. ningxiensis*	(2)
CNM20110626	soil	2002	Venezuela: Lara	6.59	6648	68.29	625	GCA_010858005.1	D	*N. yinchuanensis*	(2)
CNM20110629	soil	2002	Venezuela: Lara	6.25	5767	66.92	148	GCA_010857985.1	E	-	(2)
CNM20110639	soil	2002	Venezuela: Lara	6.21	5899	66.95	352	GCA_010868155.1	E	-	(2)
CNM20110648	soil	2002	Venezuela: Lara	6.28	5817	66.96	104	GCA_010868145.1	E	-	(2)

CNM20110649	soil	2002	Venezuela: Lara	6.26	5893	66.92	242	GCA_010868115.1	E	-	(2)
DSM 44484[T]	bronchial secretions	2000	Germany: Gelsenkirchen	6.26	5698	68.22	64	GCA_005863225.1	A	*N. cyriacigeorgica*	(1)
EML1456	infiltration basin urban sediments	2017	France	6.83	6288	68.02	108	GCA_005863295.1	C	-	(3)
EML446	infiltration basin urban sediments	2017	France	6.52	5964	68.23	41	GCA_005863245.1	C	-	(3)
GUH-2	kidney	1970s	USA	6.19	5588	68.37	1	GCA_000284035.1	D	*N. yinchuanensis*	(1, 4)
MDA3349	NA	2016	USA: Texas	6.46	5850	68.28	2	GCA_002949635.1	A	*N. cyriacigeorgica*	
MDA3732	NA	2016	USA: Texas	6.59	5980	68.22	84	GCA_002933455.1	A	*N. cyriacigeorgica*	
N18	sputum	2018	China: Guangxi	6.64	6466	68.21	484	GCA_015477345.1	A	*N. cyriacigeorgica*	
N26	sputum	2017	China: Guangxi	6.18	5695	68.42	98	GCA_015477225.1	D	*N. yinchuanensis*	
N32	sputum	2018	China: Guangxi	6.62	6022	68.22	60	GCA_015477145.1	A	*N. cyriacigeorgica*	
N43	sputum	2017	China: Fujian	6.39	5880	68.18	241	GCA_015476925.1	A	*N. cyriacigeorgica*	
N48	sputum	2015	China: Guangxi	6.07	5470	68.35	55	GCA_015476905.1	A	*N. cyriacigeorgica*	
N51	BALF	2018	China: Henan	6.79	6158	68.12	73	GCA_015476895.1	A	*N. cyriacigeorgica*	

[T] type strain.

NA: unknown.

BALF: bronchoalveolar lavage fluid.

The sequence data for newly sequenced isolates have been deposited in the GSA under accession number CRA005399.

Reference

1. Xu S, Li Z, Huang Y, Han L, Che Y, Hou X, Li D, Fan S, and Li Z, PLoS Negl Trop Dis 15:e0009665, 2021, https://doi:10.1371/journal.pntd.0009665.

2. Carrasco G, Monzón S, San Segundo M, García E, Garrido N, Medina-Pascual MJ, Villalón P, Ramírez A, Jiménez P, Cuesta I, and Valdezate S, Microorganisms 8:900, 2020, https://doi:10.3390/microorganisms8060900.

3. Vautrin F, Bergeron E, Dubost A, Abrouk D, Martin C, Cournoyer B, Louzier V, Winiarski T, Rodriguez-Nava V, and Pujic P, Microbiol Resour Announc 8:e00600-19, 2019, https://doi.org/10.1128/MRA.00600-19.

4. Zoropogui A, Pujic P, Normand P, Barbe V, Belli P, Graindorge A, Roche D, Vallenet D, Mangenot S, Boiron P, Rodriguez-Nava V, Ribun S, Richard Y, Cournoyer B, and Blaha D, BMC Genomics 14:286, 2013, https://doi:10.1186/1471-2164-14-286.
