# Supplementary material for: Comprehensive Analysis of the Nocardia cyriacigeorgica Complex Reveals Five Species-Level Clades with Different Evolutionary and Pathogenicity Characteristics
Source: mSystems. 2022 Apr 18;7(3):e01406-21. doi: 10.1128/msystems.01406-21 (PMC9239197; doi:10.1128/msystems.01406-21)
Supplement: TABLE S2 [file msystems.01406-21-s0007.pdf]

**Table S2.** Differential phenotypic properties between isolates CDC327$^{T}$, and CDC332$^{T}$ from their closely related species *N. cyriacigeorgica* DSM 44484$^{T}$.

| **Characterisitcs** | **Isolate CDC327$^{T}$** | **Isolate CDC332$^{T}$** | ***N. cyriacigeorgici* DSM 44484$^{T}$** |
|---|---|---|---|
| **Carbon source utilization (API 50 CH)** | | | |
| Glycerole | - | + | - |
| Erythritol | - | - | + |
| D-Arabinose | - | - | + |
| L-Arabinose | - | - | + |
| Ribose | - | + | + |
| L-xylose | w | - | - |
| Adonitol | w | - | - |
| β-methyl-D-xylopyranoside | - | + | + |
| Fructose | w | - | + |
| Mannose | w | - | - |
| Rhamnose | + | - | - |
| Mannitol | + | - | - |
| α-methyl-D-mannopyranoside | - | - | + |
| Lactose | - | - | + |
| D-turanose | + | - | - |
| **Enzyme activity (API ZYM)** | | | |
| Valine arylamidase | + | w | w |
| Trypsin | - | w | + |
| β-galactosidase | + | + | - |

+ positive, w weakly positive, - negative
