# Supplementary material for: Comprehensive Analysis of the Nocardia cyriacigeorgica Complex Reveals Five Species-Level Clades with Different Evolutionary and Pathogenicity Characteristics
Source: mSystems. 2022 Apr 18;7(3):e01406-21. doi: 10.1128/msystems.01406-21 (PMC9239197; doi:10.1128/msystems.01406-21)
Supplement: TABLE S3 [file msystems.01406-21-s0008.pdf]

Table S3. Major cellular fatty acid contents of CDC327^T, CDC332^T, and their closely related species *N. cyriacigeorgici* DSM 44484^T.

**Cellular fatty acid (%)**	**Isolate CDC327^T**	**Isolate CDC332^T**	***N. cyriacigeorgici* DSM 44484^T**
**Saturated acids**			
$C_{14:0}$	1.63	2.56	1.09
$C_{16:0}$	28.67	25.40	28.93
$C_{17:0}$	1.69	1.37	1.60
$C_{18:0}$	19.69	13.47	9.16
$C_{18:0}$ 10-methyl, TBSA	17.97	16.35	28.66
**Unsaturated acids**			
$C_{17:1}\omega7c$	0.52	1.01	TR
$C_{18:1}\omega9c$	4.67	2.91	3.91
**Summed features***			
3	23.66	33.04	19.23
5	-	1.04	-
9	TR	TR	1.26

TR, Trace level (<0.5%)
-, not detected

*Summed features refer to groups of two or three fatty acids that could not be separated by the Microbial Identification System. Summed feature 3 comprises $C_{16:1}\omega7c$ and/or $C_{16:1}\omega6c$; summed feature 5 comprises anteiso-$C_{18:0}$ and/or $C_{18:2}\omega6,9c$; summed feature 9 comprises $C_{16:0}$ 10-methyl and/or $C_{17:1}$ iso $\omega9c$.
