# Supplementary figures and images for: Comprehensive Analysis of the Nocardia cyriacigeorgica Complex Reveals Five Species-Level Clades with Different Evolutionary and Pathogenicity Characteristics
Source: mSystems. 2022 Apr 18;7(3):e01406-21. doi: 10.1128/msystems.01406-21 (PMC9239197; doi:10.1128/msystems.01406-21)

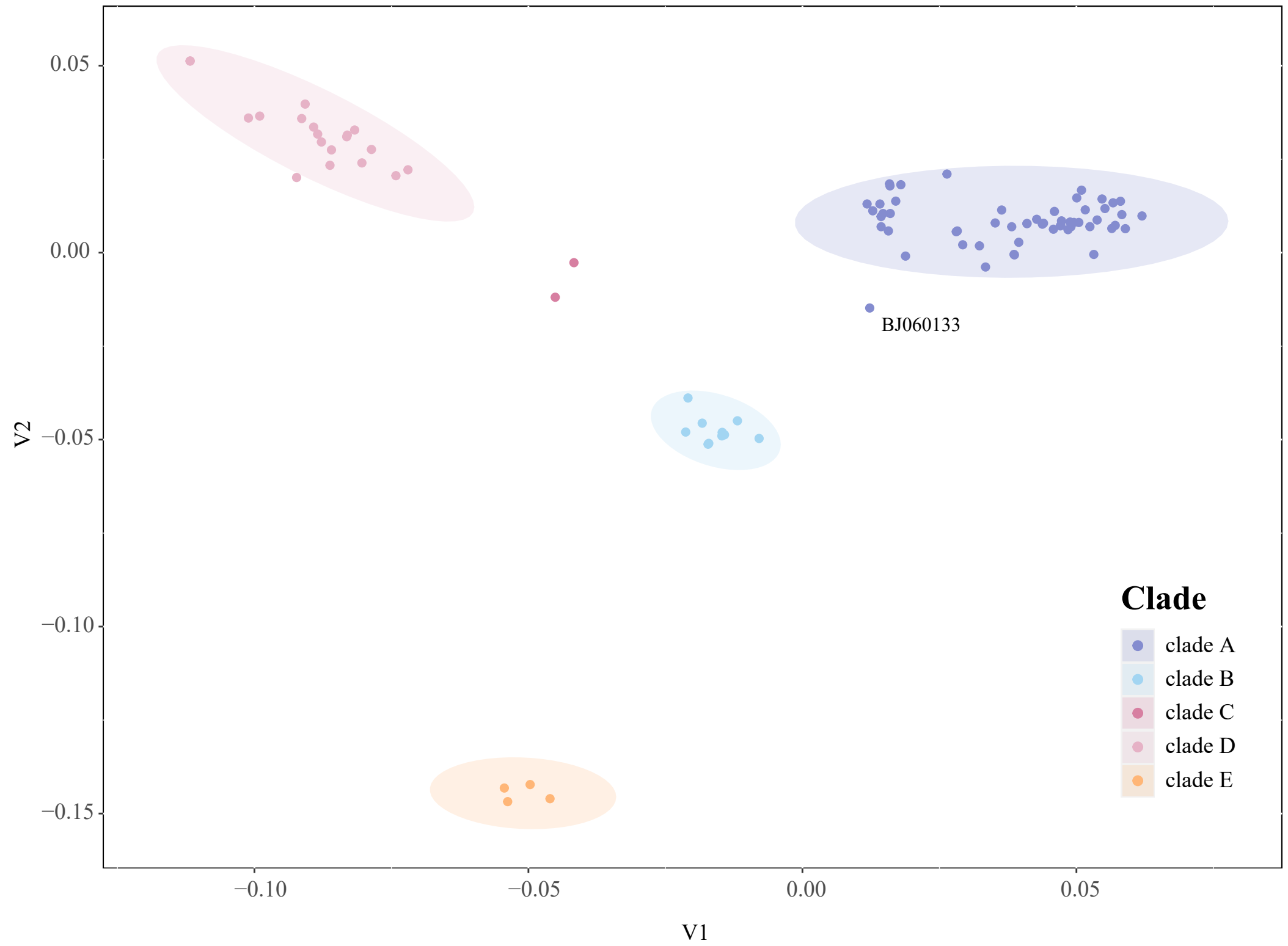

Supplement: FIG S1 [file msystems.01406-21-s0001.pdf]

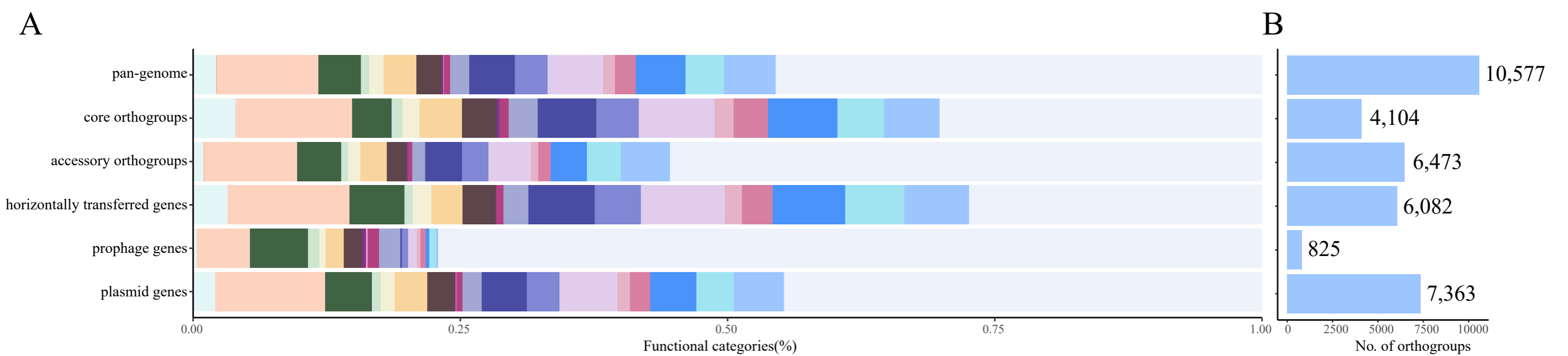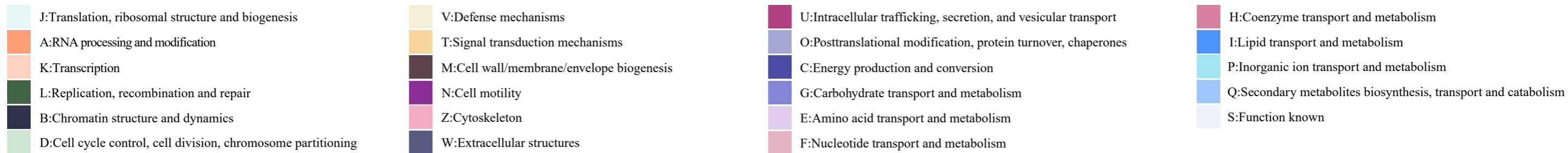

Supplement: FIG S2 [file msystems.01406-21-s0002.pdf]

No. of horizontally transferred genes

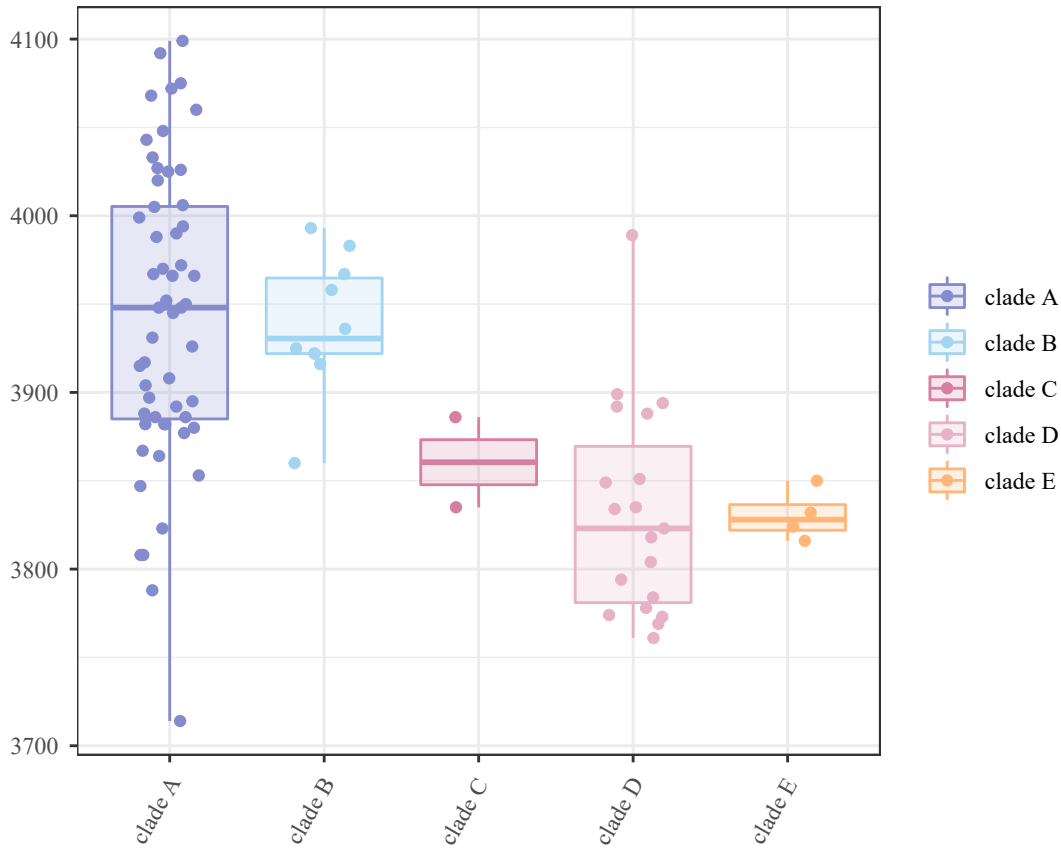

Supplement: FIG S3 [file msystems.01406-21-s0003.pdf]

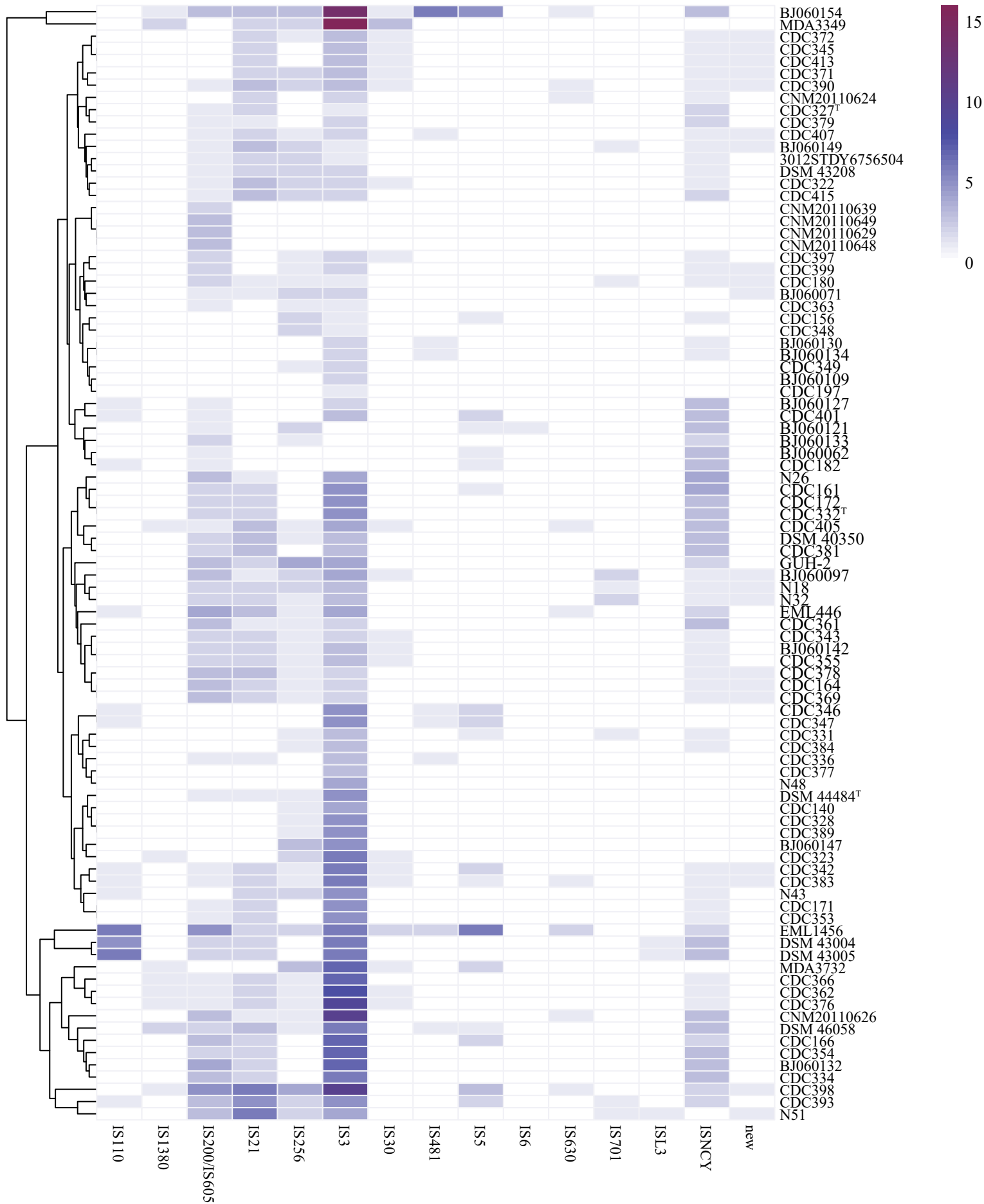

Supplement: FIG S4 [file msystems.01406-21-s0004.pdf]

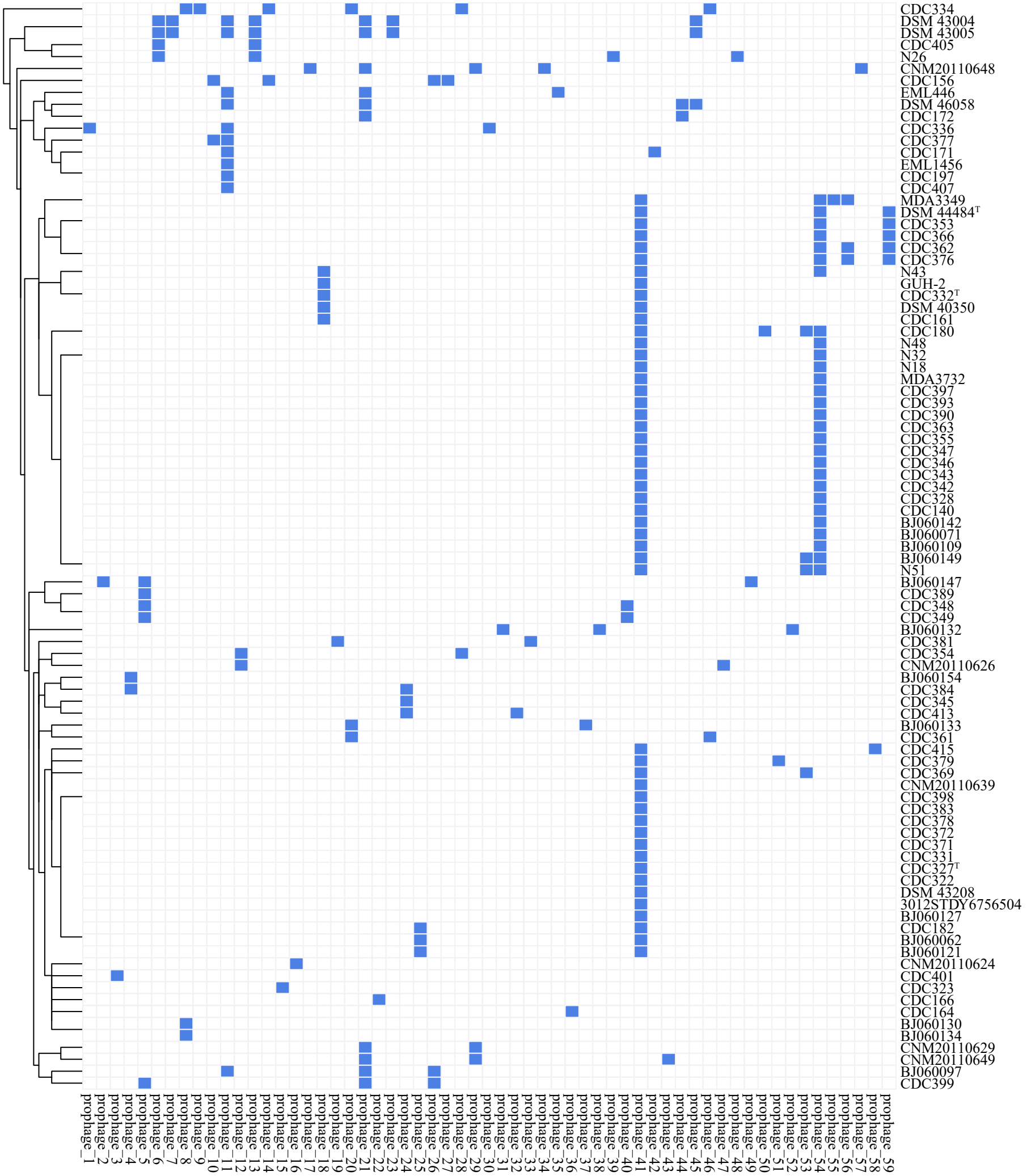

Supplement: FIG S5 [file msystems.01406-21-s0005.pdf]
