# Supplementary material for: Comprehensive Analysis of the Nocardia cyriacigeorgica Complex Reveals Five Species-Level Clades with Different Evolutionary and Pathogenicity Characteristics
Source: mSystems. 2022 Apr 18;7(3):e01406-21. doi: 10.1128/msystems.01406-21 (PMC9239197; doi:10.1128/msystems.01406-21)
Supplement: TABLE S4 [file msystems.01406-21-s0009.pdf]

Table S4. List of predicted virulence factors in the core genome.

ID	Gene name	Type	Virulence factors	Bacterial
VFG000079	clpC	Adherence and invasion	endopeptidase Clp ATP-binding chain C	Listeria monocytogenes EGD-e
VFG000077	clpP	Adherence and invasion	ATP-dependent Clp protease proteolytic subunit	Listeria monocytogenes EGD-e
VFG046465	EF-Tu	Adherence and invasion	elongation factor Tu	Francisella tularensis subsp. tularensis SCHU S4
VFG005576	eno	Adherence and invasion	phosphopyruvate hydratase	Streptococcus agalactiae A909
VFG010260	mce4D	Adherence and invasion	Mce	Mycobacterium sp. JLS
VFG016502	pdhB	Adherence and invasion	pyruvate dehydrogenase E1 component beta subunit (PDH-B)	Mycoplasma mobile 163K
VFG005358	plr/gapA	Adherence and invasion	glyceraldehyde-3-phosphate dehydrogenase (Streptococcal plasmin receptor/GAPDH)	Streptococcus pneumoniae R6
VFG042931	ppk	Adherence and invasion	polyphosphate kinase (type IV pili)	Dichelobacter nodosus VCS1703A
VFG042749	tadA	Adherence and invasion	TadA ATPase (type IV pili (AI098))	Pseudomonas aeruginosa PAO1
VFG043448	Rv1837c	Adherence and invasion	malate synthase G (Fibronectin-binding protein)	Mycobacterium tuberculosis H37Rv
VFG048830	gnd	Immune evasion	6-phosphogluconate dehydrogenase (Capsule)	Klebsiella pneumoniae subsp. pneumoniae NTUH-K2044
VFG046619	OOM_1607	Immune evasion	ribulose-phosphate 3-epimerase (Capsule)	Francisella noatunensis subsp. orientalis str. Toba 04
VFG038840	flmH	Motility	flagellar-related 3-oxoacyl-ACP reductase (Polar flagella)	Aeromonas hydrophila ML09-119
VFG045297	hisF	Endotoxin	imidazole glycerol phosphate synthase subunit HisF (LPS)	Legionella pneumophila subsp. pneumophila str. Philadelphia 1
VFG013265	orfM	Endotoxin	deoxyribonucleotide triphosphate pyrophosphatase (LOS)	Haemophilus influenzae Rd KW20

VFG013530	pgi	Endotoxin	glucose-6-phosphate isomerase (Exopolysaccharide)	Haemophilus influenzae Rd KW20
VFG045340	ricA	Secreted protein	Rab2 interacting conserved protein A (RicA)	Brucella melitensis bv. 1 str. 16M
VFG012874	ipaH	T3SS effectors	Mxi-Spa T3SS effectors controlled by MxiE	Shigella flexneri 2a str. 2457T
VFG039536	CBU_1566	T4SS effectors	Coxiella Dot/Icm type IVB secretion system translocated effector	Coxiella burnetii RSA 493
VFG049196	clpB	T6SS	protein disaggregation chaperone (T6SS-II)	Klebsiella pneumoniae subsp. pneumoniae NTUH-K2044
VFG028954	eccB4	T7SS	T7SS	Mycobacterium abscessus subsp. bolletii str. GO 06
VFG023418	eccC4	T7SS	T7SS	Mycobacterium abscessus ATCC 19977
VFG021917	espR	T7SS	T7SS	Mycobacterium smegmatis str. MC2 155
VFG012177	hlyD	Toxin	Hemolysin	Clostridium perfringens str. 13
VFG009598	narG	Anaerobic respiration	Nitrate reductase	Mycobacterium sp. JLS
VFG022803	narH	Anaerobic respiration	Nitrate reductase	Mycobacterium sp. JDM601
VFG009637	narI	Anaerobic respiration	Nitrate reductase	Mycobacterium sp. JLS
VFG022816	narK2	Anaerobic respiration	Nitrate/nitrite transporter	Mycobacterium abscessus ATCC 19977
VFG007909	ddrA	Antibiotic resistance	PDIM (phthiocerol dimycocerosate) and PGL (phenolic glycolipid) biosynthesis and transport	Mycobacterium avium subsp. paratuberculosis K-10
VFG007913	ddrA	Antibiotic resistance	PDIM (phthiocerol dimycocerosate) and PGL (phenolic glycolipid) biosynthesis and transport	Mycobacterium gilvum PYR-GCK
VFG007915	ddrA	Antibiotic resistance	PDIM (phthiocerol dimycocerosate) and PGL (phenolic glycolipid) biosynthesis and transport	Mycobacterium sp. JLS
VFG008678	ddrA	Antibiotic resistance	PDIM (phthiocerol dimycocerosate) and PGL (phenolic glycolipid) biosynthesis and transport	Mycobacterium yongonense 05-1390

VFG007929	ddrB	Antibiotic resistance	PDIM (phthiocerol dimycocerosate) and PGL (phenolic glycolipid) biosynthesis and transport	Mycobacterium sp. JLS
VFG008703	ddrB	Antibiotic resistance	PDIM (phthiocerol dimycocerosate) and PGL (phenolic glycolipid) biosynthesis and transport	Mycobacterium smegmatis JS623
VFG016307	BC5263	Antiphagocytosis	UDP-glucose 4-epimerase (Polysaccharide capsule)	Bacillus cereus ATCC 14579
VFG002182	cpsI	Antiphagocytosis	UDP-galactopyranose mutase (Capsule)	Enterococcus faecalis V583
VFG045688	uppS	Antiphagocytosis	undecaprenyl diphosphate synthase (Capsule)	Enterococcus faecium Aus0004
VFG026073	wcbT	Antiphagocytosis	8-amino-7-oxononanoate synthase	Burkholderia thailandensis E264
VFG031042	zmp1	Intracellular survival	metallopeptidase (Zn++ metallophrotease)	Mycobacterium smegmatis str. MC2 155
VFG031164	mpa	Proteasome-associated proteins	Proteasome-associated proteins	Mycobacterium intracellulare MOTT-02
VFG031121	pafA	Proteasome-associated proteins	Proteasome-associated proteins	Mycobacterium abscessus subsp. bolletii str. GO 06
VFG022829	ahpC	Stress adaptation	AhpC	Mycobacterium abscessus ATCC 19977
VFG043573	CT396	Stress adaptation	molecular chaperone DnaK	Chlamydia trachomatis D/UW-3/CX
VFG037028	katA	Stress adaptation	KatA	Neisseria meningitidis MC58
VFG037104	msrA/B pilB	Stress adaptation	trifunctional thioredoxin/methionine sulfoxide reductase A/B protein (Methionine sulphoxide reductase)	Neisseria gonorrhoeae NCCP11945
VFG043550	Rv0440	Stress adaptation	chaperonin GroEL	Mycobacterium tuberculosis H37Rv
VFG022845	sodA	Stress adaptation	sodA	Mycobacterium abscessus ATCC 19977
VFG022837	sodC	Stress adaptation	sodC	Mycobacterium abscessus ATCC 19977

VFG022664	glnA1	Amino acid and purine metabolism	Glutamine synthesis	Mycobacterium abscessus ATCC 19977
VFG026433	glnA1	Amino acid and purine metabolism	Glutamine synthesis	Mycobacterium tuberculosis RGTB327
VFG009376	leuD	Amino acid and purine metabolism	Leucine synthesis	Mycobacterium smegmatis str. MC2 155
VFG026346	proC	Amino acid and purine metabolism	pyrroline-5-carboxylate reductase	Mycobacterium smegmatis JS623
VFG026359	trpD	Amino acid and purine metabolism	anthranilate phosphoribosyltransferase	Mycobacterium tuberculosis str. Haarlem/NITR202
VFG030895	cyp125	Catabolism of cholesterol	Cyp125	Mycobacterium abscessus ATCC 19977
VFG030989	fadE29	Catabolism of cholesterol	FadE29	Mycobacterium gilvum PYR-GCK
VFG030170	caeA	Cell surface components	Carboxylesterase	Mycobacterium sp. JDM601
VFG029756	ecf	Cell surface components	GPL locus	Mycobacterium sp. JLS
VFG029760	ecf	Cell surface components	GPL locus	Mycobacterium smegmatis JS623
VFG029936	fad23	Cell surface components	GPL locus	Mycobacterium tuberculosis H37Rv
VFG029769	fadE5	Cell surface components	GPL locus	Mycobacterium smegmatis str. MC2 155
VFG030115	kefB	Cell surface components	sodium/hydrogen exchanger (Potassium/proton antiporter)	Mycobacterium sp. JLS
VFG023861	Mb2977	Cell surface components	PDIM (phthiocerol dimycocerosate) and PGL (phenolic glycolipid) biosynthesis and transport	Mycobacterium bovis AF2122/97
VFG029717	mps1	Cell surface components	GPL locus	Mycobacterium tuberculosis RGTB423
VFG029749	MSMEG_04	Cell surface components	GPL locus	Mycobacterium smegmatis str.

	04			MC2 155
VFG029504	rmlA	Cell surface components	GPL locus	Mycobacterium sp. JDM601
VFG005862	rmlB	Cell surface components	dTDP-D-glucose 4,6-dehydratase (LPS rfb locus)	Streptococcus sanguinis SK36
VFG029815	Rv0926	Cell surface components	GPL locus	Mycobacterium gilvum PYR-GCK
VFG031395	ctpV	Copper uptake	Copper exporter	Mycobacterium ulcerans Agy99
VFG031419	ctpV	Copper uptake	Copper exporter	Mycobacterium tuberculosis RGTB327
VFG044426	dahP	Iron uptake	DAHP synthetase (Vanchrobactin)	Listonella anguillarum RV22
VFG013743	fagA	Iron uptake	ABC transporter	Corynebacterium jeikeium K411
VFG013737	fagB	Iron uptake	ABC transporter	Corynebacterium jeikeium K411
VFG013731	fagC	Iron uptake	putative iron ABC transport system, ATP-binding protein	Corynebacterium jeikeium K411
VFG008180	fbpC	Iron uptake	ABC transporter	Mycobacterium vanbaalenii PYR-1
VFG021856	fxbA	Iron uptake	Exochelin	Mycobacterium smegmatis str. MC2 155
VFG013197	hemB	Iron uptake	Porphobilinogen synthase (Heme biosynthesis)	Haemophilus somnus 2336
VFG013200	hemE	Iron uptake	uroporphyrinogen decarboxylase (Heme biosynthesis)	Haemophilus somnus 2336
VFG013203	hemL	Iron uptake	glutamate-1-semialdehyde-2,1-aminomutase (Heme biosynthesis)	Haemophilus somnus 2336
VFG024069	irtB	Iron uptake	ABC transporter ATP-binding protein	Mycobacterium indicus pranii MTCC 9506
VFG029151	fadD33	Iron uptake	Mycobactin	Mycobacterium abscessus subsp. bolletii str. GO 06
VFG029181	fadE14	Iron uptake	Mycobactin	Mycobacterium tuberculosis str. Haarlem/NITR202

VFG022730	mbtB	Iron uptake	Mycobactin	Mycobacterium abscessus ATCC 19977
VFG009490	mbtE	Iron uptake	Mycobactin	Mycobacterium ulcerans Agy99
VFG001818	mbtH	Iron uptake	Mycobactin	Mycobacterium tuberculosis H37Rv
VFG026700	mbtI	Iron uptake	Mycobactin	Mycobacterium tuberculosis str. Haarlem/NITR202
VFG031277	mmpL3	Iron uptake	Heme uptake	Mycobacterium abscessus subsp. bolletii str. GO 06
VFG044286	pbtD	Iron uptake	Pyochelin	Proteus mirabilis HI4320
VFG016112	pchH	Iron uptake	Pyochelin	Pseudomonas fluorescens Pf-5
VFG044389	pdtorfF	Iron uptake	putative sulfurylase (Pyridine-2,6-dithiocarboxylic acid (PDTC))	Pseudomonas stutzeri KC
VFG044391	pdtorfH	Iron uptake	PdtorfH (Pyridine-2,6-dithiocarboxylic acid)	Pseudomonas stutzeri KC
VFG044100	phuV	Iron uptake	hemin importer ATP-binding subunit (direct heme uptake system)	Pseudomonas aeruginosa PAO1
VFG044228	PSEEN2497	Iron uptake	ABC transporter permease/ATP-binding protein (pseudomonine)	Pseudomonas entomophila L48
VFG044070	Rv0204c	Iron uptake	transmembrane protein (HasA-type hemophore-mediated heme uptake system)	Mycobacterium tuberculosis H37Rv
VFG044071	Rv0205	Iron uptake	transmembrane protein (HasA-type hemophore-mediated heme uptake system)	Mycobacterium tuberculosis H37Rv
VFG044072	Rv0207c	Iron uptake	hypothetical protein (HasA-type hemophore-mediated heme uptake system)	Mycobacterium tuberculosis H37Rv
VFG012576	sitB	Iron uptake	SitB protein (Iron/manganese transport)	Escherichia coli CFT073
VFG012586	sitD	Iron uptake	SitD protein (Iron/manganese transport)	Escherichia coli CFT073
VFG030880	cyp125	Lipid and fatty acid metabolism	Probable cytochrome P450 125 Cyp125	Mycobacterium tuberculosis H37Rv
VFG030998	fadE29	Lipid and fatty acid	Putative acyl-CoA dehydrogenase	Mycobacterium abscessus ATCC

		metabolism		19977
VFG017665	icl	Lipid and fatty acid metabolism	isocitrate lyase	Mycobacterium abscessus subsp. bolletii 50594
VFG017683	icl	Lipid and fatty acid metabolism	Isocitrate lyase	Mycobacterium tuberculosis RGTB423
VFG009135	kasB	Lipid and fatty acid metabolism	FAS-II	Mycobacterium tuberculosis RGTB327
VFG008123	panD	Lipid and fatty acid metabolism	Pantothenate synthesis	Mycobacterium tuberculosis CCDC5079
VFG009318	panD	Lipid and fatty acid metabolism	aspartate 1-decarboxylase (Pantothenate synthesis)	Mycobacterium vanbaalenii PYR-1
VFG047726	carA	Metabolic adaptation	carbamoyl phosphate synthase small subunit (Pyrimidine biosynthesis)	Francisella tularensis subsp. tularensis SCHU S4
VFG047705	carB	Metabolic adaptation	carbamoyl phosphate synthase large subunit (Pyrimidine biosynthesis)	Francisella tularensis subsp. holarctica OSU18
VFG030297	adhD	Mycolic acid synthesis	MymA operon	Mycobacterium abscessus ATCC 19977
VFG030400	fadD13	Mycolic acid synthesis	MymA operon	Mycobacterium sp. JDM601
VFG030402	fadD13	Mycolic acid synthesis	MymA operon	Mycobacterium gilvum Spyr1
VFG030203	mymA	Mycolic acid synthesis	MymA operon	Mycobacterium tuberculosis H37Rv
VFG030282	sadH	Mycolic acid synthesis	MymA operon	Mycobacterium tuberculosis RGTB327
VFG030629	sugB	Sugar transporter	sugar-transport integral membrane protein ABC transporter SugB	Mycobacterium ulcerans Agy99
VFG030724	sugC	Sugar transporter	Trehalose-recycling ABC transporter	Mycobacterium smegmatis JS623
VFG009894	devR/dosR	Regulation	two component transcriptional regulator, LuxR family	Mycobacterium vanbaalenii PYR-1

VFG009897	devR/dosR	Regulation	two component transcriptional regulator, LuxR family	Mycobacterium sp. JLS
VFG024200	devR/dosR	Regulation	DevR/S	Mycobacterium indicus pranii MTCC 9506
VFG001379	hspR	Regulation	hspR	Mycobacterium tuberculosis H37Rv
VFG022935	mprA	Regulation	two component response transcriptional regulatory protein MprA	Mycobacterium abscessus ATCC 19977
VFG022943	mprB	Regulation	two component sensor kinase MprB	Mycobacterium abscessus ATCC 19977
VFG001389	prrA	Regulation	two component response transcriptional regulatory protein	Mycobacterium tuberculosis H37Rv
VFG031723	regX3	Regulation	DNA-binding response regulator RegX3	Mycobacterium tuberculosis CDC1551
VFG031724	regX3	Regulation	two-component system response regulator	Mycobacterium leprae TN
VFG031685	senX3	Regulation	Sensor-like histidine kinase senX3	Mycobacterium abscessus ATCC 19977
VFG026975	sigA/rpoV	Regulation	RNA polymerase sigma factor	Mycobacterium tuberculosis str. Erdman (ATCC 35801)
VFG031590	sigD	Regulation	Sigma D	Mycobacterium tuberculosis H37Rv
VFG009731	sigF	Regulation	Sigma F	Mycobacterium ulcerans Agy99
VFG009732	sigF	Regulation	Sigma F	Mycobacterium smegmatis str. MC2 155
VFG009787	whiB3	Regulation	WhiB3	Mycobacterium avium 104
VFG009790	whiB3	Regulation	WhiB3	Mycobacterium smegmatis str. MC2 155
